# Microglia regulate myelin clearance and cholesterol metabolism after demyelination via interferon regulatory factor 5

**DOI:** 10.1007/s00018-025-05648-2

**Published:** 2025-03-26

**Authors:** Alejandro Montilla, Alazne Zabala, Ibai Calvo, Marina Bosch-Juan, Irene Tomé-Velasco, Paloma Mata, Mirjam Koster, Amanda Sierra, Susanne M. Kooistra, Federico N. Soria, Bart J. L. Eggen, Olatz Fresnedo, José Andrés Fernández, Vanja Tepavcevic, Carlos Matute, María Domercq

**Affiliations:** 1 Achucarro Basque Center for Neuroscience, E-48940 Leioa, Spain; 2 Department of Neuroscience, University of the Basque Country UPV/EHU, E-48940 Leioa, Spain; 3 Department of Physical Chemistry, Faculty of Sciences, University of the Basque Country UPV/EHU, E-48940 Leioa, Spain; 4 Department of Biomedical Sciences of Cells and Systems, Section Molecular Neurobiology, University Medical Center Groningen, University of Groningen, Groningen, Netherlands; 5 Ikerbasque Foundation, E-48009 Bilbao, Spain; 6 Department of Biochemistry and Molecular Biology, University of the Basque Country UPV/EHU, E-48940 Leioa, Spain; 7 Lipids & Liver Research Group, Department of Physiology, Faculty of Medicine and Nursing, University of the Basque Country UPV/EHU, E-48940 Leioa, Spain; 8 Centro de Investigación Biomédica en Red de Enfermedades Neurodegenerativas (CIBERNED), Leioa, Spain

**Keywords:** Microglia, IRF5, Multiple sclerosis, Lipid homeostasis, Demyelination, Remyelination

## Abstract

Interferon regulatory factor 5 (IRF5) is a transcription factor that plays a role in orchestrating innate immune responses, particularly in response to viral infections. Notably, IRF5 has been identified as a microglia risk gene linked to multiple sclerosis (MS), but its specific role in MS pathogenesis remains unclear. Through the use of *Irf*5^-/-^ mice, our study uncovers a non-canonical function of IRF5 in MS recovery. *Irf*5^-/-^ mice exhibited increased damage in an experimental autoimmune encephalomyelitis (EAE) model and demonstrated impaired oligodendrocyte recruitment into the lesion core following lysolecithin-induced demyelination. Transcriptomic and lipidomic analyses revealed that IRF5 has a role in microglia-mediated myelin phagocytosis, lipid metabolism, and cholesterol homeostasis. Indeed, *Irf*5^-/-^ microglia phagocytose myelin, but myelin debris is not adequately degraded, leading to an accumulation of lipid droplets, cholesterol esters, and cholesterol crystals within demyelinating lesions. This abnormal buildup can hinder remyelination processes. Importantly, treatments that promote cholesterol transport were found to reduce lipid droplet accumulation and mitigate the exacerbated damage in *Irf*5^-/-^ mice with EAE. Altogether, our study identified the antiviral transcription factor IRF5 as a key transcriptional regulator of lipid degradation and cholesterol homeostasis and suggest that loss of IRF5 function leads to pathogenic lipid accumulation in microglia, thereby obstructing remyelination. These data and the fact that *Irf5* polymorphisms are significantly associated with MS, highlight IRF5 as a potential therapeutic target to promote regenerative responses.

**Supplementary Information:**

The online version contains supplementary material available at 10.1007/s00018-025-05648-2.

## Introduction

Multiple sclerosis (MS) is a chronic inflammatory disease of the central nervous system (CNS) that leads to demyelination and axonal degeneration. It is the most common cause of non-traumatic disability in young adults [1, 2]. Although the exact mechanisms driving the onset and progression of MS remain largely unknown, inflammatory microglial activity is evident at all stages of myelin lesion development [3, 4]. Microglia, as part of the innate immune system, can both initiate and amplify inflammatory responses in MS models, leading to tissue damage [4–6]. At the same time, these cells also play a crucial role in promoting myelin repair by clearing myelin debris, known to inhibit regeneration [7, 8], and by releasing trophic and growth factors [9]. Therefore, a deeper understanding of the signaling pathways that regulate microglial activation and function is essential for fostering regenerative processes in MS.

Potential factors involved in early microglia activation comprise damage-associated molecular patterns (DAMPs), such as nucleotides, which activate purinergic receptors that regulate microglia recruitment and function [4]. Demyelinating lesions in human and mouse samples exhibit the presence of P2X4^+^ microglia, suggesting a role for purinergic signaling in lesion evolution [10]. Indeed, we have identified the purinergic receptor P2X4 as a target to modulate microglia activation and myelin phagocytosis, leading to improved remyelination in MS animal models [11]. Microglia P2X4-reactive phenotype is driven by transcription factors involved in interferon signaling such as interferon regulatory factor 5 (IRF5) [12]. IRF5 belongs to a family of nine transcription factors (IRF1-9) involved in human innate, antiviral immune responses and immune cell differentiation, with interferon (IFN) production as a hallmark of activation [13]. Among IRFs, IRF5 is a significant regulator of macrophage function, playing a key role in the induction of pro-inflammatory cytokines and chemokines, as well as promoting the pro-inflammatory phenotype of macrophages/microglia [14–16]. Therefore, we speculate that IRF5 could have an impact on demyelinating lesions evolution. The importance of IRF5 and its signaling pathway in MS pathology is further highlighted by the association of *Irf5* polymorphisms with MS susceptibility in genome-wide association studies (GWAS) [17, 18]. Polymorphisms in this gene have also been associated with an increased risk of developing and progressing neuromyelitis optica spectrum disorder (NMOSD) [19], an inflammatory autoimmune disease of the CNS that predominantly affects optic nerves and spinal cord. Furthermore, the IRF5_424–434_ peptide exhibits molecular mimicry with peptides from Epstein-Barr virus (EBV) and *Mycobacterium avium* subsp. *paratuberculosis* (MAP), two infectious agents implicated in MS pathogenesis, and induces a significant humoral response in MS patients [20]. Antibody-mediated cross-recognition of this self-epitope may alter IRF5 protein function, potentially contributing to the immune dysregulation that promotes MS development and progression [20].

Finally, IRF5 has been defined as a microglial risk gene for MS [21]. However, the role of IRF5 in microglia function and MS pathology remains mainly unknown. In this study, we demonstrate for the first time that the viral response transcription factor IRF5 is required for myelin degradation and myelin-derived cholesterol homeostasis in microglia and that it plays an essential role in recovery and remyelination. Our data suggest that IRF5’s role in microglia function could be a key factor in the progression and severity of MS.

## Materials and methods

### Human samples

Post-mortem optic nerve samples from 13 MS patients and 12 control subjects (who died from non-neurological diseases) were obtained under the management of the Netherlands Brain Bank. All patients and controls had previously given written approval for the use of their tissue, according to the guidelines of the Netherlands Brain Bank. The clinical characteristics of the different experimental groups have been previously described [22]. For comparisons, MS samples were matched with control samples for age, sex, and post-mortem delay.

### Animals

All experiments were performed according to the procedures approved by the Ethics Committee of the University of the Basque Country (UPV/EHU). Animals were handled in accordance with the European Communities Council Directive. Animals were kept under conventional housing conditions (22 ± 2°C, 55 ± 10% humidity, 12-hour day/night cycle and with *ad libitum* access to food and water) at the University of the Basque Country animal unit. All possible efforts were made to minimize animal suffering and the number of animals used. Experiments included C57BL/6 wild-type (WT) mice and *Irf5*^-/-^ C57BL/6 mice, the latter kindly provided by Prof. Tak W. Mak from the Princess Margaret Cancer Centre, UHN (Toronto, Canada).

### EAE immunization

Different EAEs were induced in 8- to 10-week-old male or female WT and *Irf5*^-/-^ mice. Mice were immunized with 200 µg of myelin oligodendrocyte glycoprotein 35–55 (MOG35–55; MEVGWYRPFSRVVHLYRNGK) in incomplete Freund´s adjuvant (IFA; Sigma) supplemented with 8 mg/mL Mycobacterium tuberculosis H37Ra (Fisher). Pertussis toxin (500 ng; Sigma) was injected intraperitoneally on the day of immunization and 2 days later, to facilitate the development of the disease model. Motor symptoms were recorded daily and scored from 0 to 8, as described elsewhere [23].

After EAE, mice were euthanized and the tissues were dissected out and differentially processed in accordance to the subsequent experimental procedure. For immunohistochemistry, the lumbar region of the spinal cord, where lesions typically accumulate, was fixed by immersion for 4 hours in 4% paraformaldehyde (PFA) dissolved in 0.1 M phosphate buffer (PB, pH = 7.4), rinsed in phosphate-buffered saline (PBS) and then transferred to 15% sucrose in 0.1 M PB for at least 2 days for cryoprotection. Next, tissue was frozen in 15% sucrose - 7% gelatine solution in PBS, and cut in a Leica CM3050 S cryostat to obtained 12-µm coronal sections. For real-time quantitative polymerase chain reaction (qPCR), the cervical and thoracic regions of the spinal cord, as well as peripheral immune-related organs, such as spleen or lymph nodes, were flash frozen in dry ice. For flow cytometry, the whole spinal cord was isolated.

### Lysolecithin-induced demyelination

To analyze remyelination in *Irf5*^-/-^ mice, we performed lysolecithin-induced demyelination in the spinal cord of both WT and knock-out male mice. The lesions were induced by stereotaxic injection of 0.5μL of 1% lysolecithin (LPC; Sigma) in saline solution, as previously described [24, 25]. Briefly, animals were anesthetized by intraperitoneal injection of a solution of ketamine (100 mg/kg) and xylazine (10 mg/kg). The tissue covering the vertebral column was making two longitudinal incisions into the *longissimus dorsi*, and the intravertebral space of the 13^th^ thoracic vertebra was exposed by removing the connective tissue after fixing the animal in the stereotaxic frame. Dura mater was then pierced using a 30G needle, and LPC was injected via a Hamilton syringe attached to a glass micropipette using a stereotaxic micromanipulator.

The lesion specific site was marked with sterile charcoal so that the area of tissue at the center of the lesions could be unambiguously identified afterwards. Following LPC injection, the wound was sutured and mice were allowed to recover. Mice were euthanized 4 and 14 days after surgery, in order to assess the response to demyelination. After LPC-induced demyelination, mice were perfused with 2% PFA for 15–20 minutes and spinal cords were post-fixed in 2% PFA for another 30 minutes. Tissue was then processed the same way as EAE lumbar spinal cords.

### Immunofluorescence

Coronal sections of spinal cords from control animals, EAE mice and mice with LPC demyelinating lesions as well as microglia *in vitro* were analyzed by immunohistochemistry (IHC) or immunocytochemistry (ICC). Primary antibodies used for immunofluorescence on these samples include: mouse anti-myelin basic protein (MBP) (1:1000; #808401 BioLegend), rabbit anti-MBP (1:200; #AB980 Millipore), rabbit anti-Iba1 (1:500; #019–19741 Wako Chemicals), mouse anti-SMI32 (1:1000; #801701 BioLegend) rat anti-CD3 (1:50; #MCA1477 Bio-Rad), rat anti-CD45R (1:200; #557390 BD Bioscience), mouse anti-iNOS (1:100; # 610329 BD Biosciences), mouse anti-GFAP (1:40; #MAB3402 Millipore), mouse anti-Olig2 (1:1000; # MABN50 Millipore), mouse anti-APC (1:200; # OP80 Millipore), mouse anti-IRF5 (1:200; # ab181553 Abcam), mouse anti-ABCA1 (1:100; # Ab18180 Abcam) and mouse anti-ABCG1 (1:100; #Ab218528 Abcam). These primary antibodies were subsequently detected by incubation with appropriate Alexa Fluor 488, 594 or 647 conjugated goat antibodies (1:250; Invitrogen). Cell nuclei were stained using Hoechst 33258 (Sigma-Aldrich).

For Oil red O staining of EAE and LPC-induced demyelinating lesions, manufacturer’s protocol was followed. Briefly, tissues were incubated with 60% isopropanol for 5 minutes followed by a staining step with 60% Oil Red O dissolved in isopropanol for 15 minutes. Excess of stain was then rinsed with distilled water and IHC was performed subsequently.

Images were acquired using a Leica TCS STED SP8 confocal microscope, a Zeiss LSM800 confocal microscope or a Pannoramic MIDI II slide scanner (3DHistech) with the same settings for all samples within one experimental group. For the visualization of cholesterol crystals, confocal reflection microscopy was performed on the tissues using a ZEISS LSM 800 Airyscan confocal microscope, as these structures strongly reflect the light of the excitation laser. All the image analysis was performed with the ImageJ software (National Institutes of Health; NIH).

For histological analysis of EAE lesions, images of the whole section were obtained. Lesion extents, as well as axonal damage, were normalized to the total white matter (WM) area of each section. The lesion area was defined by the lack of MBP staining along with the accumulation of myelin debris (identified by an increase in MBP fluorescence), and the accumulation of Iba1^+^ cells was normalized to this lesioned extent. At least three sections were analyzed per animal. For phagocytosis analysis, we automatically detected myelin blobs using the Threshold tool with a Gaussian blur (radius=1) and a Yen threshold. Microglia were identified using the Threshold tool with Yen threshold, and the Analyze particles tool to fill in the phagocytic pouches. Next, we quantified phagocytosis as the % blobs within microglia. For the assessment of LPC-induced lesions, dorsal funiculus images were obtained using a 20x objective, and analyses were performed similarly to those in the EAE tissue. To evaluate the distribution of different cell types in relation to the demyelinating lesions, we analyzed the fluorescence intensity of the markers in radial profiles comprising both lesioned and non-lesioned WM using the “Lesion_profiler v.1.1” script (https://github.com/SoriaFN/Tools). After manual ROI creation to delineate the lesions, profiles were automatically drawn with a fixed width (250 px) and length (1 diameter of lesion ROI). Fluorescence intensity was averaged between profiles and normalized to allow comparisons. Distance was normalized likewise.

For ICC experiments, mean fluorescence intensity (fluorescence intensity/cell area, as defined in ImageJ) of IRF5, ABCA1 and ABCG1 was calculated in individual cells, defined on the basis of Iba1 immunostaining (data was obtained from 20–30 cells per coverslip from 3–5 different experiments performed in duplicate). Regarding IRF5, the mean intensity (IRF5 fluorescence/ROI area) was calculated in defined ROIs in cytoplasm and nucleus and the results were expressed as the ratio (mean fluorescence in cytoplasm/mean fluorescence in nucleus).

### Quantitative RT-PCR

Total RNA from EAE lumbar spinal cords, spleens and lymph nodes as well as from control and MS post-mortem optic nerve samples was isolated using TRIzol (Invitrogen) following the manufacturer's instructions. Afterwards, 2 µg of this RNA was used to perform a retrotranscription protocol, using SuperScript III Reverse Transcriptase (200 U/μL; Invitrogen) and random hexamers as primers (Promega).

qPCRs were conducted using a Bio-Rad Laboratories CFX96 real-time PCR detection system, using iTaq Universal SYBR Green Supermix (Bio-Rad), that includes SYBR Green as DNA-binding dye and iTaq DNA polymerase. The specific primers for different T cell subtypes were designed Primer Express software (Applied Biosystems) at exon junctions to avoid genomic DNA amplification. The cycling conditions comprised 3 min of polymerase activation at 95°C and 40 cycles consisting of 10 s at 95°C and 30 s at 60°C. The amount of cDNA was quantified using a standard curve from a pool of cDNA obtained from the different conditions of the experiment. Finally, the results were normalized using a normalization factor based on the geometric mean of housekeeping genes obtained for each condition using the geNorm v3.5 software [26].

### RNA sequencing

Bulk RNA-sequencing was performed on total microglial populations, isolated by fluorescence-activated cell sorting (FACS), from control spinal cords of WT and *Irf5*^-/-^ mice. In order to isolate the cells while maintaining their specific activation state, all sorting steps were performed at 4 ºC. Spinal cords were mechanically dissociated and nuclear cells were isolated from debris using a Percoll gradient. Single cell suspensions were incubated with TruStain FcX™ (anti-mouse CD16/32) antibodies for 15 minutes to block unspecific bindings, and then stained for 30 minutes with CD11b-FITC (1:200; #101206 BioLegend), CD45-PE (1:100; #103106 BioLegend), Ly6C-PE/Cy7 (1:300; #128017 BioLegend) and SYTOX AADvanced™ Ready Flow™ (#R37173 Thermo Fisher), a viability marker. We identified microglial population as SYTOX^-^/CD11b^+^/CD45^low^/Ly6C^-^. Cells were collected in RNAprotect Cell Reagent (Qiagen), and total RNA was extracted using the RNeasy Plus Micro kit (Qiagen), following manufacturer’s instructions.

NEBNext Low Input RNA Library Prep Kit for Illumina was used to process samples (n = 4 and 3 for WT and *Irf5*^-/-^ microglia, respectively) (GenomeScan, Leiden, The Netherlands). RNA concentration and quality was determined with a Fragment Analyzer. Next, cDNA was synthesized and amplified from poly-A tailed mRNA. Clustering and DNA sequencing using the NovaSeq6000 was performed according to manufacturer's protocols, using a concentration of 1.1 nM DNA. At least 20 million paired-end reads were generated per sample, with a quality score of ≥ 30. Quality checks, reads trimming and alignment to the most recent mouse genome were also performed.

All downstream bioinformatic analyses were performed in RStudio (v2021.09.0). For the differential gene expression analysis, low and non-expressed genes were excluded. The Bioconductor package edgeR [27] (v3.34.1) was used for normalization using the timed mean of M-values (TMM) method, and for identification of the differentially expressed genes (DEGs) between the different experimental groups by fitting a generalized linear model. DEGs were identified as those with an adjusted p-value < 0.05 and a log (Fold Change) > 1. Gene ontology (GO) analysis of the recognized DEGs for every comparison was performed using the DAVID [28] and Metascape [29] web resources.

*Irf5* expression was analysed in scRNAseq sequencing data from Meijer *et al.* [30] and from Zhang *et al.* [31]. Data visualization and interactive analysis were performed using the UCSC Cell Browser [32]. For in silico quantification of *Irf5*, raw data obtained from Gene Expression Omnibus (accession number: GSE124335) was analyzed [33]. This data corresponds to single-cell RNA sequencing results, coming from active MS lesions as well as control tissues. Specifically, Tmem119^+^ cells were selected for the quantification.

### MALDI-MS

In order to evaluate the changes in the lipidomic signatures in the context of LPC-induced demyelination, 12 µm-thick coronal sections were obtained from WT and *Irf5*^-/-^ mice spinal cords at 14 dpi. Tissues were scanned using a MALDI-LTQ-Orbitrap XL (Thermo Fisher) in the Spectroscopy Unit of the University of the Basque Country (UPV/EHU), using the negative-ion mode for the m/z region where the most relevant lipid species appear (650–1200 Da). The sections were covered with a 1,5-diaminonaphtalene matrix [34], using an in-house designed sublimator, and introduced in the MALDI source. Data acquisition was performed with a spatial resolution of 100 µm/pixel and 60.000 at m/z = 400 mass resolution.

For the processing of the lipid signatures obtained, the obtained spectra were processed using a software developed in MatLab (MathWorks). Briefly, the peaks obtained were identified and filtered. Then, the different lipid signatures related to the diverse regions of the spinal cord were segmented using a k-means clustering method. The lipidomic profile of the LPC-induced lesion, as well as the peri-lesion area and the healthy white matter of 5 WT and 5 *Irf5*^-/-^ injected mice were extracted and subsequently compared.

### Primary microglia culture

Primary mixed glial cultures were prepared from the cerebral cortex of neonatal mice (P0-P6). After 10–15 days in culture, microglia were isolated by mechanical shaking (400 rpm, 1 h) and purified by plating them on non-coated bacterial grade Petri dishes (Thermo Fisher Scientific), as previously described [35]. Microglial cells obtained through this method were cultured in DMEM (Gibco) supplemented with 10% FBS (Gibco), at different cellular densities in accordance with the following experimental procedure. These cultures were practically pure of microglial cells ( > 99%) [36].

### Myelin phagocytosis assay

Mouse myelin was isolated as previously described [37]. Briefly, spinal cord was mechanically homogenized in 0.32 M sucrose and subjected to repeated sucrose gradient centrifugation and osmotic shocks to separate myelin from other cellular components. Myelin concentration was measured with Bradford assay and adjusted to 1 mg/mL. Then, myelin was labelled with Alexa488-NHS dye (A2000 Life Technologies) for 1 hour at RT in PBS (pH 8). Dyed myelin was dialyzed to remove dye excess and resuspended in PBS (pH 7.4).

For the assessment of microglial phagocytosis, myelin was vortexed for 60 seconds for fragmentation and added to microglia culture medium (5 μg/mL). To evaluate myelin endocytosis, WT and *Irf5*^-/-^ primary microglia were incubated with Alexa488-NHS-labeled myelin for 1 hour at 37ºC, rinsed and immediately fixed with 4% PFA. To evaluate myelin degradation, microglial cells were incubated with this myelin for 1 hour, rinsed and fixed 24 hours later. Myelin was quantified on Iba1^+^ cells using ImageJ on individual microglial cells outlined with the Iba1 immunostaining as the defining parameter for the ROIs. At least 50 cells were analyzed from each experiment (*n* = 3 independent experiments performed in duplicates).

### Wound healing assay

In order to assess the migratory capacity of WT and *Irf5*^-/-^ microglia, cells were seeded in DMEM + 10% FBS in glass-bottom dishes (Ibidi), generating a confluent monolayer. The monolayer was scratched in a straight line using a sterile 200 mL pipette tip. To follow the migration of microglia towards the scratched area, we performed a 24-hour time-lapse of the cells using a BioStation IM-Q microscope (Nikon), maintaining the dishes at 37ºC and 5% CO_2_ during the whole extent of the experiment. The percentage of the scratched area occupied by microglia was quantified in the initial image obtained, as well as in images after 12 and 24 hours.

### Lipid extraction and quantification

In order to assess lipid metabolism of WT and *Irf5*^-/-^ microglia, we challenged these primary cells with 25 μg/mL purified myelin for 48 hours. After treatment, excess of myelin was washed with PBS, and cells were subsequently scrapped and centrifuged. Cell pellets were resuspended in PBS and sonicated in 2 cycles of 10 seconds, with intervals of 10 seconds between cycles and 25% amplitude. For lipid extraction, a commonly used method was carried out [38]. Briefly, 2 mL of chloroform and 4 mL of methanol were added to 40 µg of protein from the cell homogenates; this initial volume also included a mixture of lipid standards. Tubes were vigorously shaken for 2 minutes, and 2 mL of chloroform were then added to the mixture. After shaking for another minute, 3.2 mL of distilled water was added to the tubes, and another 1-minute vortex step was performed. This mixture was centrifuged at 1500 g for 10 minutes, at 4ºC, to allow the separation of the aqueous and organic phases. The lower, organic phases containing the lipids were transferred to clean tubes. Lipids retained in the aqueous phase were re-extracted by adding a mixture of chloroform, methanol and distilled water and repeating the shaking and centrifuge steps, and the new organic phase was combined with the previously obtained one. Last, the solvent was evaporated using a Thermo Savant SC250 EXP SpeedVac vacuum concentrator to obtain the final lipid extract.

Lipids in this extract were analyzed using UltiMate 3000 ultrafast liquid chromatography system (UHPLC; Thermo Scientific) coupled to a QExactive™ HF-X Hybrid Quadrupole-Orbitrap mass spectrometer (MS), in the University of the Basque Country (UPV/EHU) facilities. The extract was resuspended in 90 µL of 9:1 methanol:toluene mixture, and 7 µL of the resulting supernatant was injected into the HPLC-MS system. Electrospray ionization was performed in either positive or negative ion mode. Lipid species predicted by the quantification of the HLPC-MS results were filtered, classified into families and quantified in accordance to the standard signals and their concentrations. Lipid ontology (LION) analysis of the differential lipids was performed using the LION/web application [39]. This experiment was performed on *n* = 4 different cultures from WT and *Irf5*^-/-^ mice, and the data represent the mean quantity (µg) of each species in a lipid class, normalized to the quantity of initial protein from each sample.

### Statistical analysis

Data are presented as mean ± standard error of mean (SEM) with the sample size and number of repeats indicated in the figure legends. Statistical analyses were performed using GraphPad Prism 8 (GraphPad Software Inc). Comparisons between two groups were analysed using paired Student’s two-tailed t-test for data coming from *in vitro* experiments, unpaired Student’s two-tailed test for data coming from *in vivo* experiments and Mann-Whitney U test in the case of comparisons regarding EAE neurological scores. Comparisons among multiple groups were analysed by one-way ANOVA followed by Bonferroni post-hoc analysis. In all instances, p values < 0.05 were considered as statistically significant.

## Results

### IRF5 deficiency exacerbates damage at EAE recovery phase

**Fig. 1:**
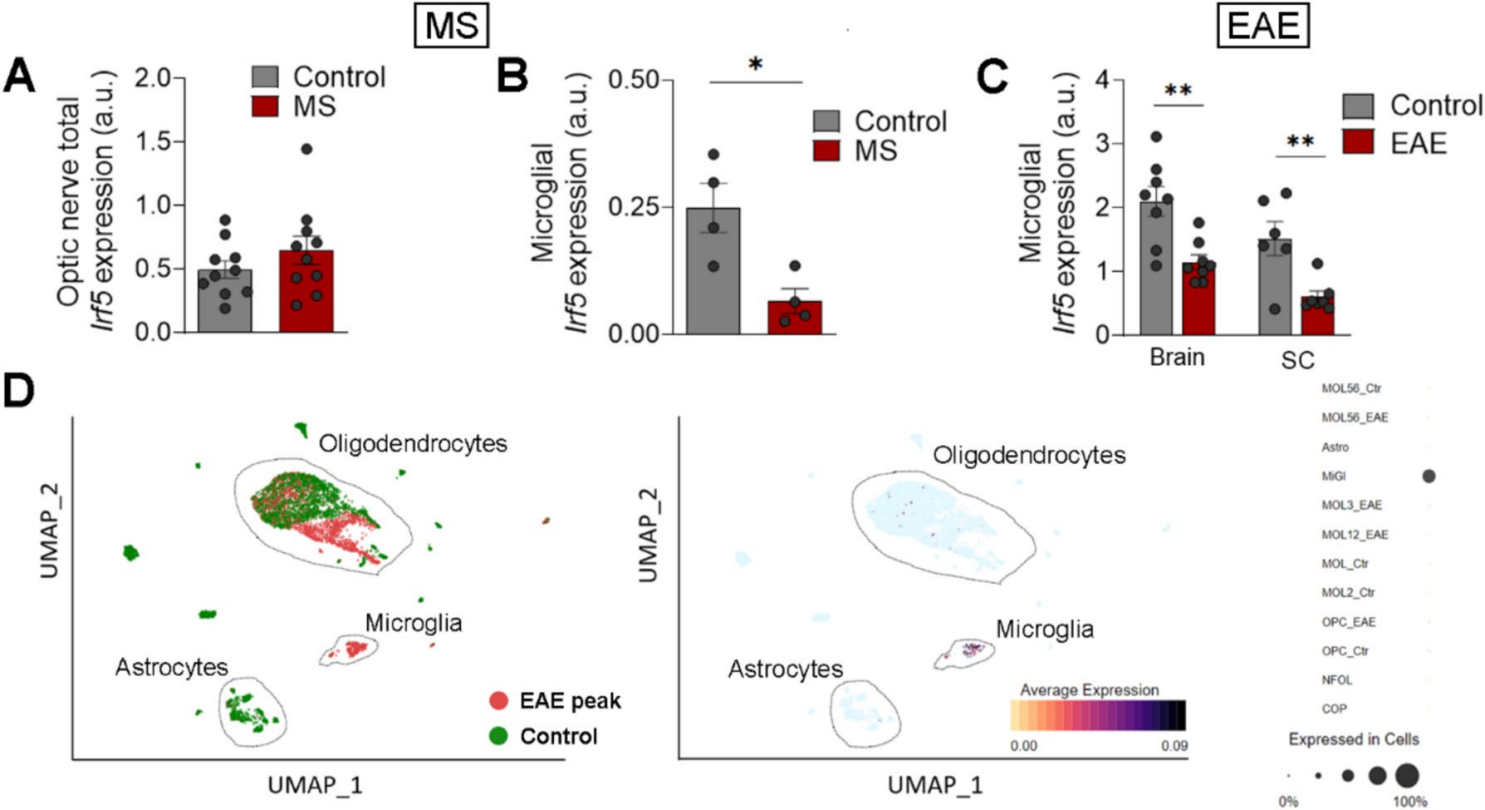
Microglial *Irf5* expression is decreased in MS patients. **A** Relative expression of *Irf5* in total mRNA isolated from post-mortem optic nerve samples of control and MS patients (*n* = 10) as analyzed by qPCR. **B** *In silico* quantification of microglial *Irf5* expression in healthy and early active MS human brain tissues (*n* = 4). Raw data obtained from Gene Expression Omnibus (accession number: GSE124335). Data are presented as mean ± SEM and were analyzed by Student's t‐test (A, B, D) and by one-way ANOVA (C). *p < 0.05, **p < 0.01, ***p < 0.001. **C** Relative expression of *Irf5* in FACS isolated microglia (CD11b^+^/CD45^low^) from the brain and spinal cord of control mice and from EAE mice at chronic phase (*n = 6–8*). **D** Reanalysis of *Irf5* expression from scRNAseq data obtained at EAE peak [31, 33]

*Irf5* is a transcription factor whose expression increases during inflammation and plays a key role in pro-inflammatory activation of microglia, contributing to their P2X4^+^ reactive state [12, 14]. This state is linked to enhanced myelin phagocytosis and remyelination in the EAE model [11]. To address whether *Irf5* expression is altered during demyelination in human, we assessed its expression in tissues coming from MS patients. We did not detect differences in the expression of IRF5 transcription factor in total RNA from post-mortem optic nerve samples of control and MS patients [23], as analyzed by qPCR, (Fig. 1A), suggesting that this gene is not upregulated during the development or at late stages of the disease. However, an *in silico* analysis of available data obtained by single-cell RNA sequencing of tissues coming from healthy humans and active multiple sclerosis patients [33] showed that *Irf5* is downregulated in microglial cells in the pathology (Fig. 1B; *n* = 4). We further analyzed microglia specific expression of Irf5 after EAE immunization. Whereas an increase in *Irf5* expression was detected in total spinal cord RNA both at EAE peak and chronic phase [11], the specific expression of *Irf5* in FACS-isolated microglia (Cd11b^+^CD45^high^) decreased at EAE (Fig. 1C). *Irf5* is expressed almost exclusively in microglia in CNS parenchyma (Fig. 1D, Sup. Fig.1A), and upregulation of *Irf5* in total RNA after EAE correlated with an increase in the microglia/infiltrating macrophage population, as evidenced by the increased expression of their classical markers Iba1 and Cd11b (Sup. Fig. 1B). Thus, the global increase in *Irf5* expression probably reflects an increase in the number of cells expressing this transcription factor, rather than a change at single-cell level.

**Fig. 2:**
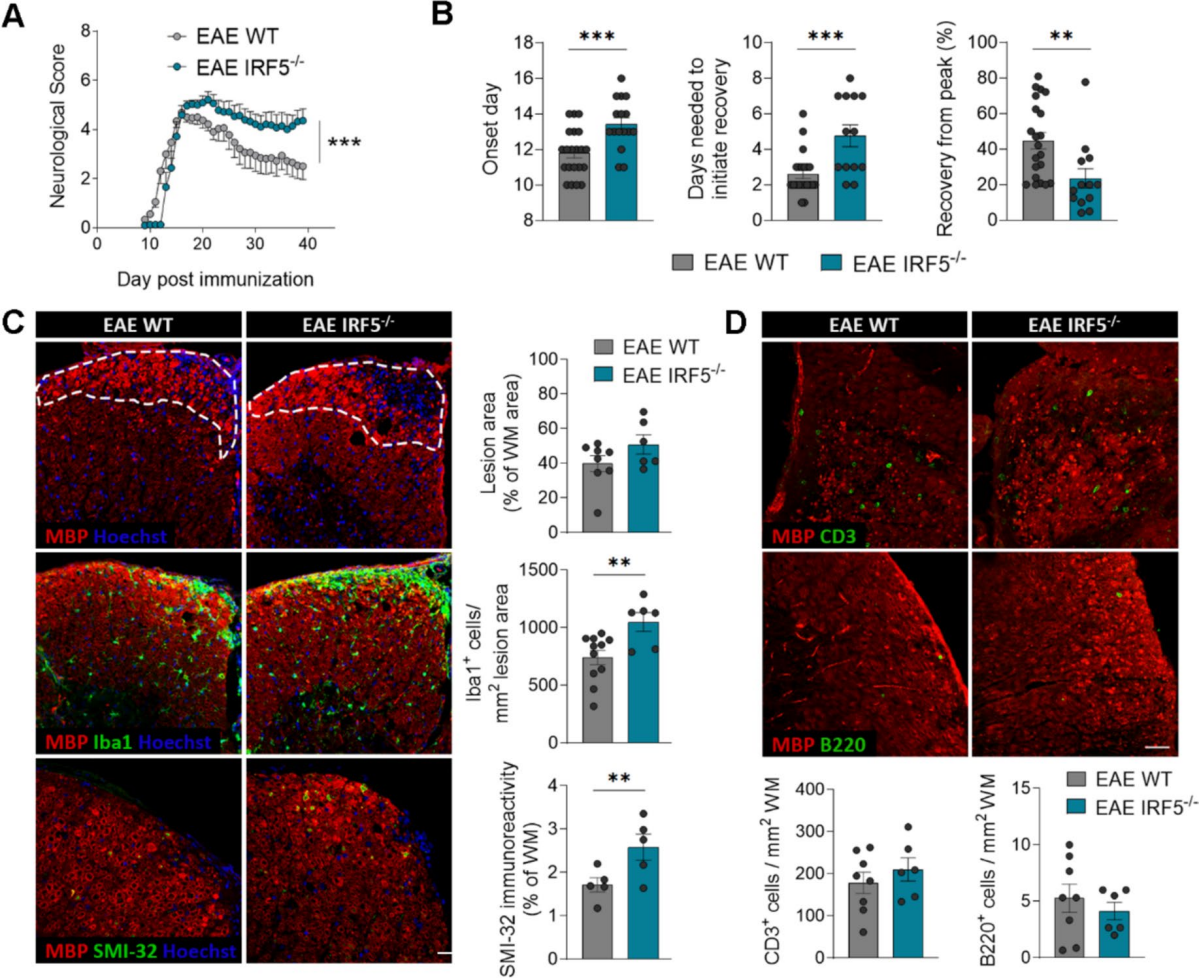
IRF5 deficiency exacerbates EAE recovery phase. **A** Neurological score of WT and *Irf5*^-/-^ mice (n = 10 mice per group; one representative experiment of three independent experiments). **B** Histograms showing clinical parameters associated to EAE induction (onset day) and recovery (days needed to initiate recovery and percentage of recovery from peak), in WT and *Irf5*^-/-^ mice (*n* = 13–20). **C** Representative images of lumbar spinal cord EAE lesions (top), Iba1 (middle) and SMI32 staining’s (bottom) in WT and *Irf5*^-/-^ mice. Immunohistochemistry was performed at 40 post-immunization. Scale bar = 30 µm. Histograms show the extent of the lesions in relation to the total white matter area of the section analyzed (*n* = 6–8) and the accumulation of Iba1^+^ microglia/macrophages (*n* = 6–11) as well axonal damage (SMI-32) in relation to the lesioned area or the total white matter area, respectively (*n* = 5). **D** Representative images showing the accumulation of CD3^+^ T cells and B220^+^ B cells in EAE lesions of WT and *Irf5*^-/-^ mice at day 40 post-immunization. Scale bar = 30 µm. Histograms show the number of cells normalized to the white matter area (*n* = 6–8). Data are presented as means ± SEM. Statistics were performed with Mann-Whitney U test (neurological score, A) and Student's t–test (B-D). **p < 0.01, ***p < 0.001.

We then addressed the impact of *Irf5* deletion on EAE progression. We observed a significant delay in the onset of motor symptoms in *Irf5*^-/-^ mice compared to WT ones (Fig. 2A, B), suggesting a role of this transcription factor in peripheral immune priming, as previously stated for other IRF transcription factors [40]. Despite this initial delay, *Irf5*^-/-^ mice showed no difference in the maximal neurological score at EAE peak (Fig. 2A, B). However, *Irf5*^-/-^ mice presented exacerbated neurological scores at EAE chronic phase (Fig. 2A, B) and showed an increase in the time necessary to initiate recovery (Fig. 2B), suggesting that IRF5 may be needed for EAE recovery. At histological level, demyelinated lesions, defined by the presence of myelin loss or damage, tended to be larger although this change was not statistically significant (Fig. 2C; p = 0.074). However, we found a higher accumulation of Iba1^+^ cells inside the lesions and an increase in axonal damage (assessed with SMI32 marker) in *Irf5*^-/-^ mice (Fig. 2C). These observations confirm the exacerbation of tissue damage in *Irf5*^-/-^ mice at EAE chronic phase.

Since IRF5 is a transcription factor involved in immune response, the worsening of neurological symptoms in the chronic phase of EAE could be linked to changes in this response. To test this hypothesis, we analyzed the immune response at EAE chronic phase. We did not observe any significant difference in the accumulation of T cells or B cells in the lesions, assessed by CD3 and B220 staining respectively, at EAE chronic phase (Fig. 2D). To delve into the immune response, we also measured the levels of CD4^+^ and CD8^+^ cells in the spinal cord, as well as the different CD4^+^ T cell subtypes in spinal cord and peripheral immune organs, by qPCR. We did not find significant alterations in the expression of *Cd4* or *Cd8* (Fig. S2A), nor in the expression of *FoxP3, Ror* and *Ifnγ*, signature genes for Treg, Th17 and Th1 cells, respectively (Fig. S2B). These results suggest that the differences observed in EAE chronic phase are not due to alterations in the adaptive immune response. As IRF5 is known to be involved in regulating microglia/macrophage response [14, 15], we next analyzed whether *Irf*5 deletion could shift microglia activation. The expression of different pro-inflammatory and anti-inflammatory genes in microglia was upregulated in *Irf5*^-/-^ mice at EAE chronic phase, as analyzed by Fluidigm qPCR (Fig. S2C), correlating with an increase in Iba1 and Cd11b (Fig. S2D), but no significant shift was detected in the pro- versus anti-inflammatory profiles. Altogether, these data suggest that IRF5 transcription factor is necessary to EAE recovery through a mechanism different from its classic modulatory role of the immune response.

### IRF5 deficiency worsens LPC-induced demyelination and alters oligodendrocyte recruitment

**Fig. 3:**
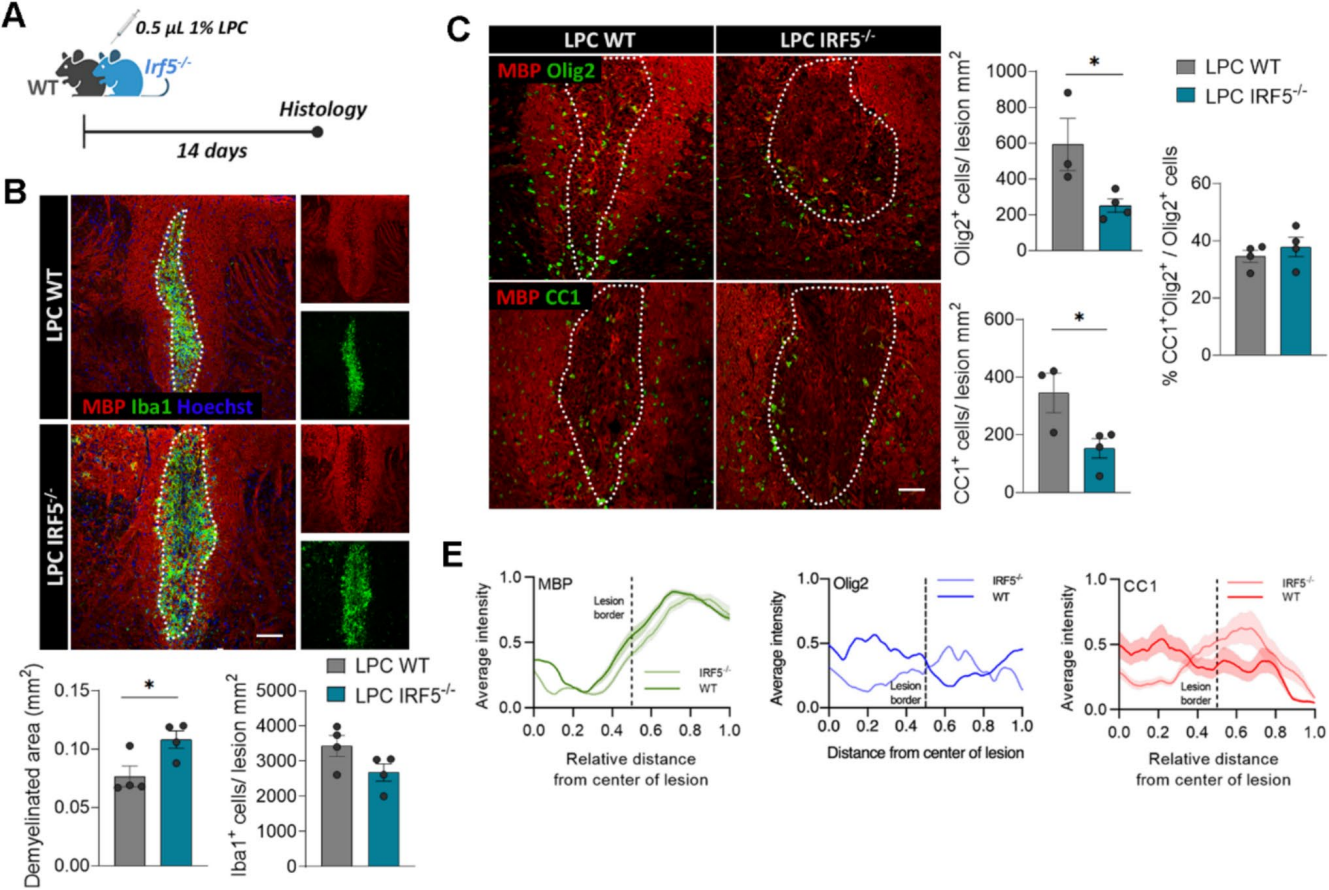
IRF5 deficiency alters remyelination after lysolecithin (LPC)-induced lesions. **A** Scheme showing the experimental design of LPC-induced demyelinated lesions. Analysis was performed at 14 post-injection, a time coincident with the initiation of the remyelinating response.** B** Representative images of MBP and Iba1 stainings in LPC lesions of WT and *Irf5*^-/-^ mice. Scale bar = 75 µm. Histograms show the extent of demyelinated area and the accumulation of microglia in the lesions in each mice (*n* = 4). **C** Assessment of the number of Olig2^+^ and CC1^+^ oligodendrocytes in LPC-induced lesions, delineated by MBP loss (*n* = 3–4). Scale bar = 75 µm.** D** Distribution analysis (mean ± SD) of MBP (left), Olig2 (middle) and CC1 (right) immunostaining, in an area comprising equal distances of lesioned and non-lesioned white matter (lesion border indicated with dotted lines; *n* = 4). Note the higher accumulation of Olig2^+^ and CC1^+^ cells outside the lesion core. Data are presented as means ± SEM. Statistics were performed with Student's t-test. *p < 0.05

To confirm that IRF5 is essential for remyelination, as suggested by our EAE data, we employed a chemically induced demyelination model. This model, in which demyelination does not involve the immune system, is more suitable for studying the mechanisms of remyelination [24]. We induced focal demyelinating lesions in wild-type (WT) and *Irf5*^*-/*-^ mice by injecting 1% lysophosphatidylcholine (LPC) into the white matter tracts of the spinal cord. The lesions were histologically analyzed 14 days later (Fig. 3A), when oligodendrocyte precursor cells (OPCs) had been recruited into the lesions, differentiated into mature oligodendrocytes, and begun the remyelination process [24].

In accordance with the EAE results, *Irf5*^-/-^ mice showed an exacerbated pathology upon LPC injections, presenting larger lesions (Fig. 3B) at 14 days post-injection. Moreover, *Irf5*^*-/*-^ mice presented more abundance of infiltrating CD3^+^ T cells in the lesions at 14 days post-injection (Fig. S3A), indicative of an aberrant or exacerbated inflammatory and immune response in response to myelin damage. In spite of these results, we did not detect more axonal damage as determined by SMI32 immunostaining (Fig. S3B). Regarding the remyelination process, we detected a decrease in the total number of oligodendrocytes, determined by the Olig2 marker, in the lesions of *Irf5*^-/-^ mice (Fig. 3C). Similarly, we found a diminished population of mature oligodendrocytes in these animals, assessed by CC1 staining (Fig. 3C); nevertheless, the proportion of myelinating CC1^+^ Olig2^+^ cells in relation to the total Olig2^+^ population was not different between genotypes (Fig. 3C). This points out to an alteration in oligodendrocyte recruitment, and not in their differentiation capacity. This feature is accompanied by an abnormal distribution of oligodendrocytes within the lesions. Both Olig2^+^ and CC1^+^ oligodendrocytes were mainly disposed in the lesion border and peri-lesion in *Irf5*^*-/*-^ mice, rather than in the lesion core delineated on the basis of MBP immunoreactivity loss (Fig. 3C and Fig. 3D), suggesting impaired recruitment of oligodendrocytes into the lesion core in *Irf5*^*-/-*^ mice. Altogether, these findings corroborate that IRF5 is necessary for a proper remyelination response.

### Transcriptional profiling of *Irf5*^*-/*-^ microglia shows alterations in metabolism and intracellular signaling

**Fig. 4:**
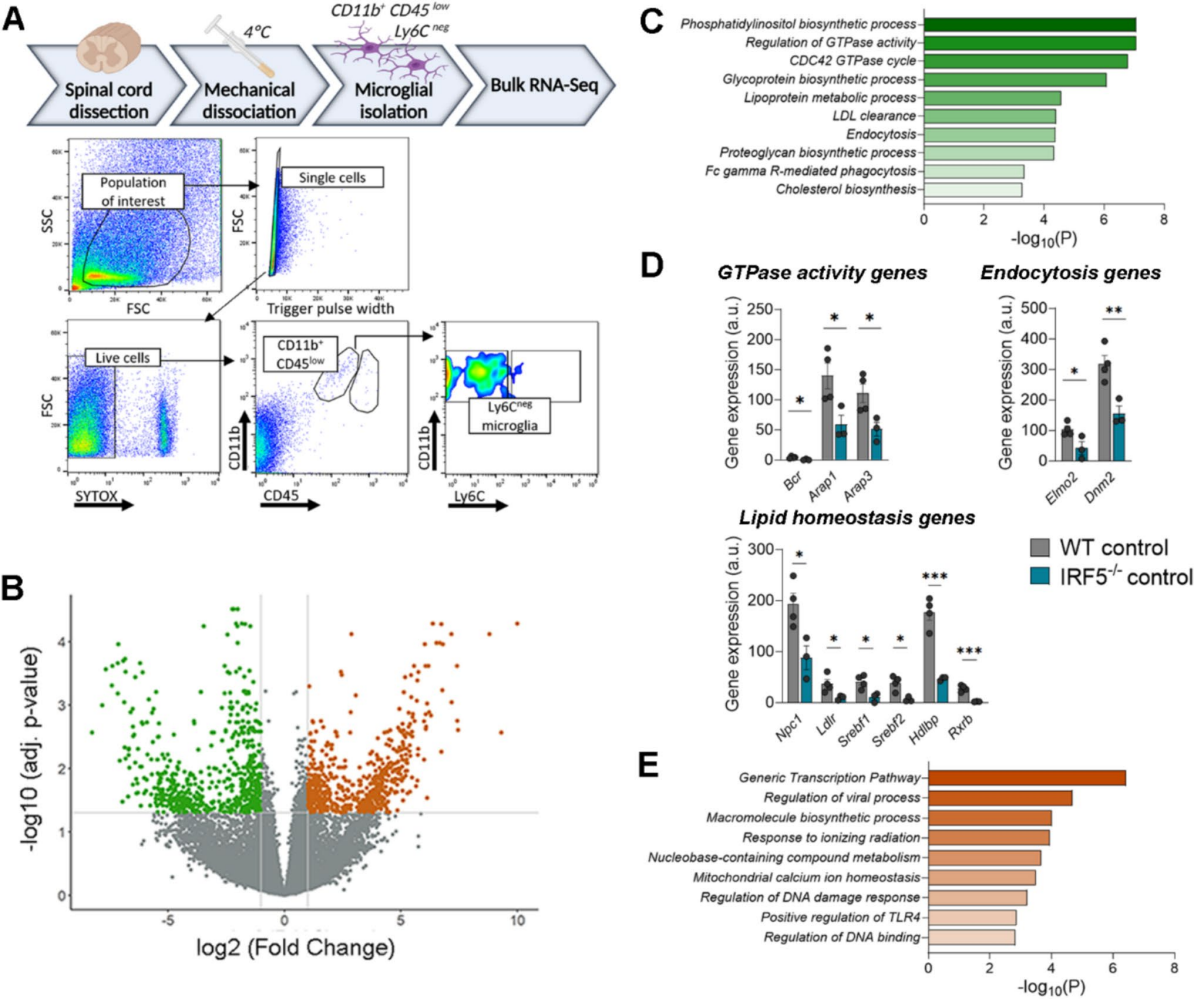
RNA sequencing of WT and *Irf5*^-/-^ microglia highlights novel roles for IRF5. **A** (Above) Experimental strategy for isolating spinal cord microglia at 4 ºC to avoid overactivation and RNA sequencing. (Below) Flow cytometry gating strategy for isolation of microglia from the spinal cord of WT and *Irf5*^-/-^ mice. **B** Volcano plot depicting gene expression comparison between WT and *Irf5*^-/-^ microglia. Each dot represents an individual gene. Non-significant genes are marked in gray while significant ones (log_2_ (FC) > 1 and p-value < 0.05) are marked in colour. **C** GO enrichment analysis of the DEGs identified between WT and *Irf5*^-/-^ microglia, showing the top GOs enriched in WT condition. Green plot shows annotations downregulated in *Irf5*^-/-^ microglia. **D** Histograms showing alterations in genes involved in GTPase activity (top left), endocytosis (top right) and lipid homeostasis (bottom) (*n* = 3–4). **E** GO enrichment analysis of the DEGs identified between WT and *Irf5*^-/-^ microglia, showing the top GOs enriched in KO condition. Orange plot shows annotations downregulated in *knock-out* microglia. Data are presented as means ± SEM. Statistics were performed with Student's t–test.*p < 0.05

Since microglia play a critical role in successful remyelination [41, 42], and IRF5 regulates microglia responses [15, 16], we performed bulk RNA sequencing on FACS-sorted microglia to identify signaling pathways regulated by Irf5 in these cells. We FACS-sorted microglia (Cd11b^+^ CD45^low^ Ly6C^-^ population; gating strategy in Fig. 4A) from the spinal cords of control WT and *Irf5*^*-/*-^ mice (Fig. 4A). We detected a high number of differentially expressed genes between WT and *Irf5*^*-/*-^ mouse microglia (Fig. 4B; DEGs had a log (Fold Change) > 1 and adjusted p-value < 0.05). GO enrichment analysis revealed that genes downregulated in *Irf5*^-/-^ microglia are associated with GTPases signaling, such as “Regulation of GTPase activity” or “CDC42 GTPase cycle” (e.g., *Bcr*, *Arap1*, *Arap3),* but mostly linked to metabolism and specifically to lipid metabolism, including “Phosphatidylinositol biosynthetic process”, “Lipoprotein metabolic process”, “LDL clearance” or “Cholesterol biosynthesis” (e.g., *Npc1*, *Ldlr, Srebf1/2*, *Hdlbp, Rxrb*...). Moreover, we also observed an association of these downregulated genes with “Endocytosis” or “Fc gamma R-mediated phagocytosis” (e.g., *Elmo2*, *Dnm2)*. All these transcriptional alterations observed in *Irf5*^-/-^ microglia could potentially be the root for the unsuccessful remyelination in mice lacking IRF5 (Fig. 4C, D). Predictably, other enriched GOs were related to inflammation and specific immune responses (not shown).

Conversely, IRF5-deficient microglial cells upregulated genes associated with specific immune responses such as “Regulation of viral processes” (e.g., *C3*) or “Positive regulation of TLR4” (e.g., *Wdfy1*) (Fig. 4E); this is expected to be linked to the well-known role of IRF5 in immunity. Moreover, these cells also upregulated pathways associated with DNA transcription or response to DNA damage, such as “Generic Transcription Pathway”, “Response to ionizing radiation” “Regulation of DNA damage response”, “Regulation of DNA binding” or even “Macromolecule biosynthetic process” (e.g., *Bcl2*, *Brf2* or different zinc finger proteins) (Fig. 4E). This outcome could be explained on the basis of the described pro-apoptotic and pro-cell cycle arrest functions of IRF5, which also participates in the p53 pathway [43, 44].

The transcriptional profiling of *Irf5*^-/-^ microglia highlights the relevance of this transcription factor in these cells. It revels its modulatory role in intracellular pathways beyond the expected immune responses and in processes that are crucial for successful remyelination.

### Irf5^-/-^ microglia show reduced motility *in vitro*

GTPases, particularly the Rho family of small GTPases (Rho, Rac, and Cdc42), play a crucial role in regulating the dynamic organization of the intracellular actin cytoskeleton. As a result, their pathways can affect a wide range of functions, such as cellular motility and phagocytosis [45, 46]. Indeed, Cdc42 signaling is essential for both microglial migration and the phagocytosis of degenerating neurons [47]. Given the observed downregulation of GTPase pathways in *Irf5*^-/-^ microglia compared to WT cells, we hypothesized that these functions might be compromised.

**Fig. 5:**
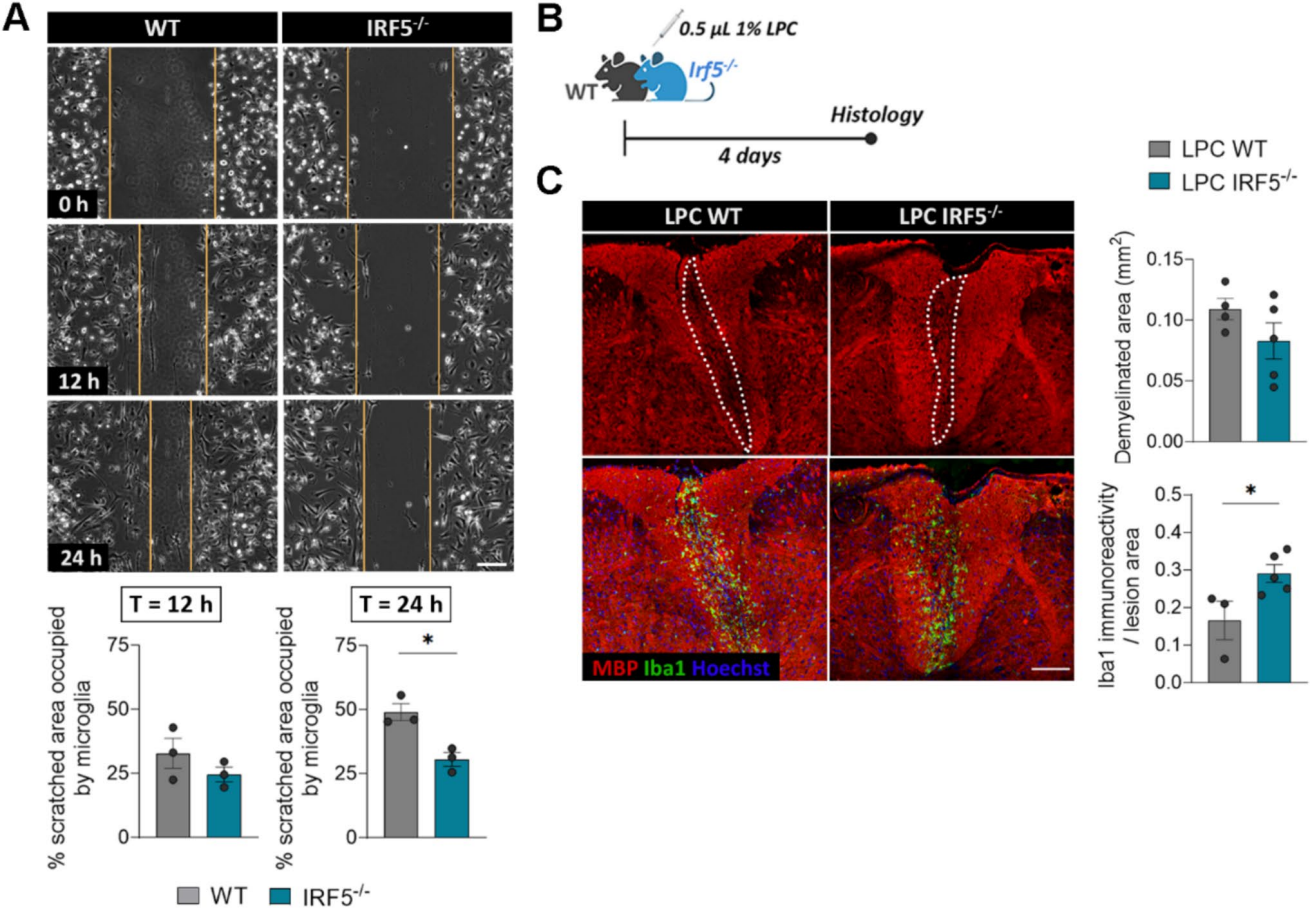
*Irf5*^*-/-*^ microglia showed altered motility *in vitro* but not after demyelination. **A** Representative frames of the wound healing assay performed on WT and *Irf5*^-/-^ microglia, at the initial time of the experiment as well as after 12 and 24 hours. Yellow lines delimitate the scratched, non-occupied area at each timepoint. Scale bar = 10 µm. Histograms below show the percentage of the initially scratched area occupied by microglial cells (*n* = 3 independent experiments). **B** Scheme showing the experimental design for the histological analysis of microglial migration after LPC demyelinating lesions, in WT and *Irf5*^-/-^ mice, at day 4 post-injection. **C** Representative confocal images of LPC-induced lesions 4 days after injection, showing MBP and Iba1 immunostaining. Scale bar = 100 µm. Histograms show the extent of demyelinated area in WT and *Irf5*^-/-^ mice and Iba1^+^ immunoreactivity in relation to the lesioned area in each animal (*n* = 4–5). Data are presented as means ± SEM. Statistics were performed with Student's t–test.*p < 0.05

First, we performed wound healing assays on WT and *Irf5*^-/-^ cultured microglia to test their migratory capacity and found that, after 24 hours, IRF5-deficient microglia were less efficient than WT cells repopulating the scratched area (Fig. 5A). This highlights that the altered GTPases signaling in *Irf5*^-/-^ microglia lead to abnormal motility *in vitro*, a fact that could affect microglial response to demyelination *in vivo*. Indeed, macrophages lacking other member of this family, IRF8, cannot migrate toward the epicenter of spinal cord lesions and remain widely scattered [48]. However, we did not detect major changes in microglial migration towards the EAE or LPC lesions at the chronic phase (30–35 days for EAE and 14 days for LPC; see Fig. 2C and 3B). To further check the impact of IRF5 deficiency on microglial migration towards demyelinating lesions, we histologically analyzed LPC-induced lesions at 4 days post-injection, a time point coincident with microglia/macrophage arrival (Fig. 5B) [24]. At this stage, there was no significant difference in the arrival of Iba1^+^ microglia/macrophage migration into the lesions of WT and *Irf5*^-/-^ mice. Rather, *Irf5*^-/-^ mice showed more Iba1 immunoreactivity in the demyelinated areas than WT mice did at this timepoint (Fig. 5C). Moreover, at this timepoint, there was no difference in the extent of demyelinated area between WT and *Irf5*^-/-^ mice (Fig. 5C), suggesting no differences at initial demyelination responses after LPC (Fig. 5C).

These data suggest that, although *Irf5*^-/-^ microglia showed some reduced motility *in vitro*, this deficit does not appear to be responsible for the changes observed in the demyelinating models. Additionally, since the LPC-induced lesions showed no differences between genotypes at early stages, this points out to secondary mechanisms influencing later remyelination events.

### IRF5 deletion alters myelin clearance both *in vivo* and *in vitro*

Myelin clearance from the lesions by myeloid cells is essential for an efficient regenerative response [9, 49]. Since RNA sequencing revealed downregulation in the endocytic and phagocytic pathways in *Irf5*^-/-^ microglia, we next analyzed whether deficiencies in microglial phagocytosis of myelin could contribute to the regeneration failure observed in *Irf5*^-/-^ mice.

**Fig. 6:**
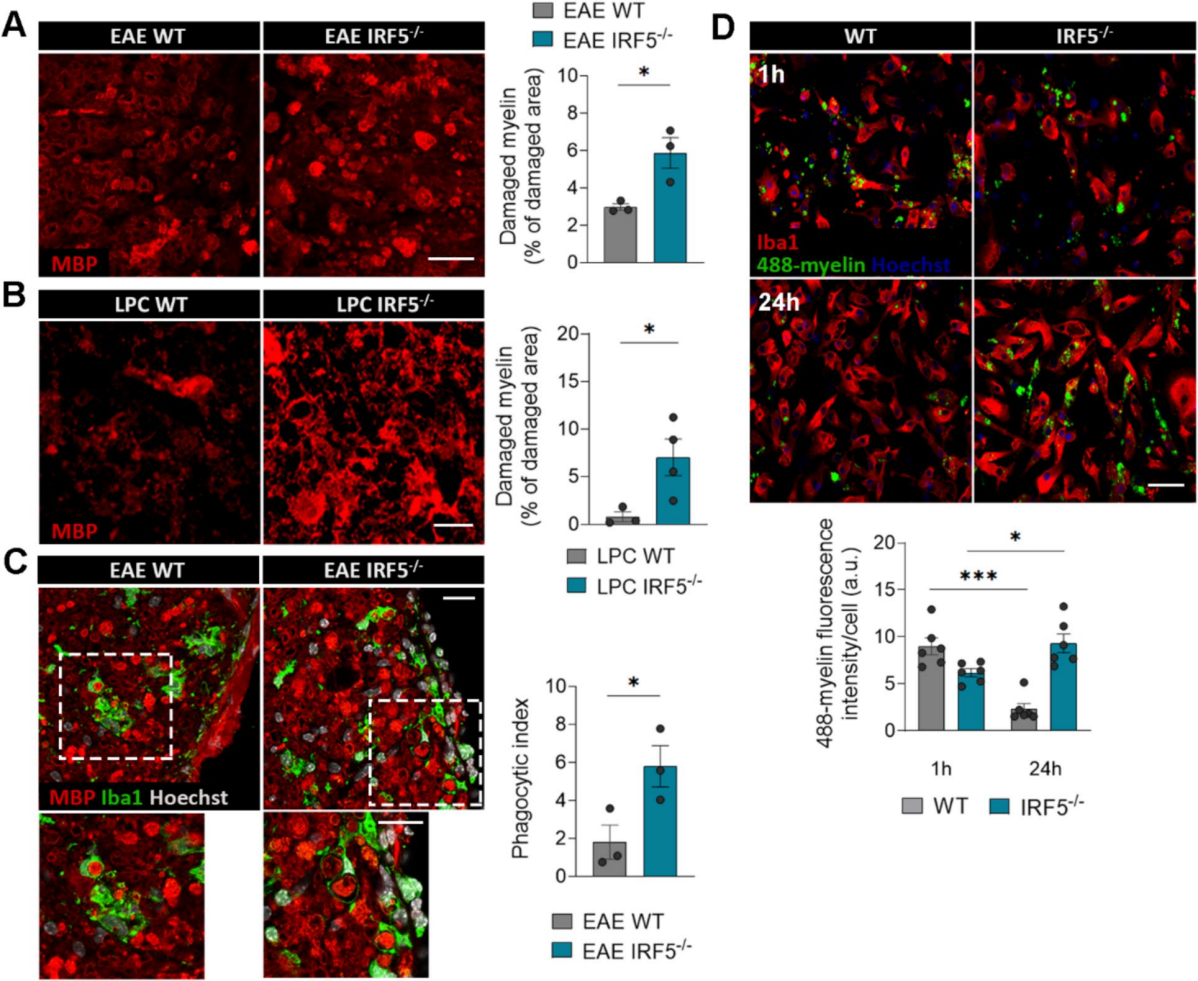
Myelin phagocytosis and degradation are altered in *Irf5*^*-/-*^ microglia both *in vitro* and after demyelination.** A** Representative images of myelin debris accumulation (characterized by high MBP immunoreactivity) in WT and *Irf5*^-/-^ EAE lesions, at the recovery phase. Scale bar = 50 µm. Histogram shows the lesioned area occupied by this debris in each section (*n* = 3). **B** Representative images of myelin debris accumulation at day 14 post-LPC demyelinating injections, in WT and *Irf5*^-/-^ mice. Scale bar = 10 µm. Histogram shows the lesioned area occupied by this debris in each animal (*n* = 3–4). **C** Representative images of MBP and Iba1 immunostaining in spinal cord sections of WT and *Irf5*^-/-^ mice. Insets show higher magnifications of the indicated boxes. Scale bar = 20 µm. Histogram shows the phagocytic index of microglia/macrophages in these conditions (*n* = 3). **D** Representative images showing phagocytosis (1h) and degradation (24h) of Alexa-488 labelled-myelin by WT and *Irf5*^-/-^ microglia *in vitro*. Scale bar = 50 µm. Histogram shows the fluorescence of 488-myelin in the cells, defined as ROIs using Iba1 staining (*n* = 6 independent experiments). Data are presented as means ± SEM. Statistics were performed with Student's t–test. *p < 0.05, ***p < 0.001

First, we analyzed whether IRF5-deficient animals accumulated more myelin debris after demyelination. Indeed, *Irf5*^-/-^ mice showed a higher accumulation of disrupted or fragmented myelin both in EAE chronic phase and 4 days after LPC injections in the spinal cord (Fig. 6A, B). Damaged myelin yields higher MBP immunoreactivity due to the unmasking of protein epitope [11]. Moreover, we quantified myelin phagocytosis by microglia/macrophage in EAE lesions, assessing the presence of MBP^+^ debris inside Iba1^+^ ROIs that include the whole cytoplasm, processes and pouches using custom Image J macros. *Irf*5^-/-^ mice showed a higher phagocytic index (% of blobs within microglia), meaning a higher accumulation of myelin debris in microglia cells pouches or cytoplasm (Fig. 6C). We observed that myelin debris size was bigger and preferentially located in the phagocytic processes of *Irf5*^-/-^ Iba1^+^ cells. In contrast, WT microglia presented more abundance of partially degraded myelin in the cytoplasm (Fig. 6C). Thus, although *Irf5*^-/-^ microglia showed an increase in myelin phagocytosis or accumulation, the differential size and distribution of myelin debris may indicate an impairment in myelin degradation after endocytosis.

To further explore myelin phagocytosis, myelin was isolated from adult mouse whole brain using sucrose gradient [37], labelled with the dye Alexa-488 and added to microglia cultures. In order to efficiently clear up myelin, microglia should internalize myelin and deliver it to lysosomes to degrade it. We monitored by confocal microscopy myelin engulfment (1h) and myelin degradation later on (24h). We observed a significant decrease in myelin engulfment in *Irf5*^-/-^ microglia after 1h (Fig. 6D). Moreover, while WT microglia properly degraded the internalized myelin after 24 hours, *Irf5*^-/-^ microglia showed a faulty degradatory process (Fig. 6D).

These findings suggest that IRF5 deficiency is linked to alterations in myelin phagocytosis and/or degradation, which could be the underlying cause of the regeneration failures observed in *Irf5*^-/-^ mice in response to demyelination.

### IRF5 deficiency impairs lipid homeostasis and myelin metabolism after demyelination

For debris clearance to be effective towards repair, microglia must also process their internalized cargo and dispose of it appropriately [8, 9, 50, 51]. Microglia can degrade myelin debris at lysosomes into sterols that help resolve inflammation. However, myelin also causes an overload of cholesterol, which must be released through specific transporters or stored in lipid droplets to avoid toxicity [52]. The transcriptional profile of *Irf5*^-/-^ microglia reveals alterations in lipid metabolism, which could explain the differing responses to demyelination in animals lacking this transcription factor. In fact, genes involved in lipid endocytosis (*Ldlr*), lipids egress from lysosomes (*Npc1*), cholesterol efflux (*Hdlbp*), and the transcriptional regulation of lipid homeostasis (*Srebf1* and *Srebf2*) were downregulated in *Irf5*^-/-^ microglia (Fig. 4D). These latter genes play a role in generating sterols, which serve as ligands for liver X receptors (LXRs), promoting regenerative actions [53]. Notably, the expression of the gene encoding for retinoid X receptor beta (*Rxrb*), which dimerizes with LXRs and peroxisome proliferator-activated receptors (PPARs), was also downregulated in *Irf5*^-/-^ microglia (Fig. 4D). All these genes are critical modulators of lipid and cholesterol metabolism, and deficiencies in some of them are associated with lipid related pathologies [54]. This suggests that lipid homeostasis could be impaired in *Irf5*^-/-^ microglia.

To gain deeper insight into lipid metabolism in *Irf5*^-/-^ microglia following demyelination, we conducted MALDI-MS imaging alongside immunohistochemistry on the same LPC- induced demyelinated spinal cords. After MALDI imaging, the tissues were immunolabeled with antibodies against myelin basic protein (MBP) and ionized calcium-binding adapter molecule 1 (Iba1) to delineate the lesions and define regions of interest (ROI), including normal white matter, lesion periphery, and lesion core. MALDI segmentation identified three main regions: normal white matter, lesion core, and lesion periphery. The lesion periphery, which is more enriched with microglia/macrophages, could help us delineate specific changes in these cell populations. We then analyzed the MALDI spectra and associated lipid signatures corresponding to these regions (Fig. S4A). When comparing the lipid profiles of healthy white matter and lesion core, we found a significant decrease in ceramides, sulfatides, plasmalogens, and phosphatidylserines (Fig. S4B)—characteristic lipids of myelin [37]—in the lesion cores of both WT and *Irf5*^-/-^ lesions. Since myelin is rich in these lipids [55], this reduction likely indicates demyelination. Additionally, we observed differences in the accumulation of phosphatidylcholines and phosphatidylethanolamines (data not shown), which are major components of cellular membranes. This increase in phosphatidylcholines and phosphatidylethanolamines could correlate with inflammatory processes [56].

The primary lipid changes observed at the periphery of LPC-induced lesions showed intermediate values between healthy white matter and completely demyelinated white matter in the lesion core (Fig. S4C), likely reflecting the ongoing demyelination process. Although we did not find significant differences in lipid profiles between WT and *Irf5*^*-/-*^ mice in normal white matter or in the lesion core, there was a trend toward lower levels of sulfatides and higher levels of phosphatidylcholines in the lesion periphery in *Irf5*^*-/-*^mice (Fig. S4D). This may suggest distinct responses to demyelination between the genotypes. However, it is unclear whether these changes are related to demyelination itself or to the way microglia and macrophages metabolize myelin.

**Fig. 7:**
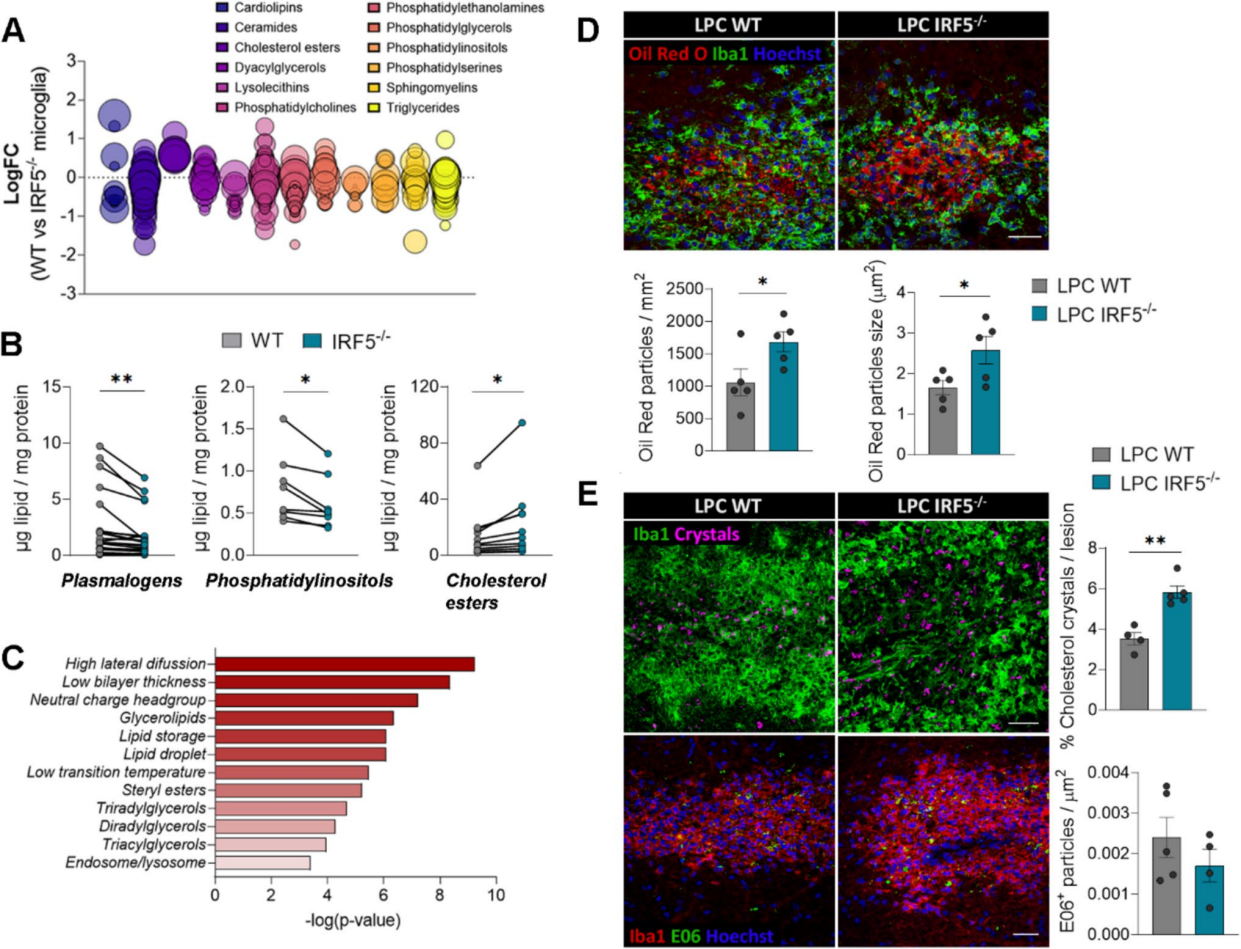
IRF5 deficiency leads to defective myelin processing and accumulation of abnormal lipid structures.** A** Bubble plot showing the concentration differences of specific lipids, classified by lipid classes, accumulated in WT and *Irf5*^-/-^ microglia, after 48 hour-treatment with myelin (*n* = 4 independent experiments). Each bubble represents a unique lipid species, and the size of the bubbles represents the significance of the individual lipid comparison. **B** Histograms showing the concentration of different plasmalogens, phosphatidylinositols and cholesterol esters in WT and *Irf5*^-/-^ microglia, after 48 hours of myelin challenge. Data are presented as means for every lipid species from 4 independent experiments. **C** Lipid ontology (LION) enriched terms in *Irf5*^-/-^ microglia compared to WT microglia. **D** Immunostaining of Oil Red O (ORO) and Iba1 in LPC-induced lesions (14 dpi) in WT and *Irf5*^-/-^ mice. Histograms show the number and size of ORO^+^ particles in the lesions (*n* = 5). Scale bar = 25 µm. **E** Representative images of cholesterol crystals, acquired by reflection microscopy (above), and E06^+^ (below) particles in LPC-induced lesions (14 dpi), both in WT and *Irf5*^-/-^ mice. Histograms show the percentage of lesioned area occupied by crystals and the number of EO6^+^ particles normalized to the lesion area (n = 4–5). Scale bar = 30 µm. Data are presented as means ± SEM. Statistics were performed with Student's t-test. *p < 0.05, **p < 0.01

To more accurately analyse the impact of IRF5 in myelin metabolism in microglia, we challenged both WT and *Irf5*^-/-^ cultured microglia with an excess of myelin (25 µg/mL) for 48 hours, and then intracellular lipids were isolated and measured by HPLC-MS. We observed clear differences in how myelin was processed between the two groups (Fig. 7A, B). Specifically, in IRF5-deficient microglia, there were significant reductions in the concentrations of various phospholipid families, including plasmalogens and phosphatidylinositols (Fig. 7B). Additionally, we observed a significant increase in the levels of all cholesterol ester (CE) species in these cells (Fig. 7B), while total free cholesterol levels showed no difference between the groups (data not shown). Lipid ontology (LION) enrichment analysis suggested an upregulation of lipid storage in droplets, likely related to the detected increase in CE levels (Fig. 7C). The LION analysis also indicated an accumulation of lipids with high lateral diffusion that form thinner bilayers (Fig. 7C), suggesting increased membrane dynamics. Other enriched terms were associated with endosomal/lysosomal lipids and glycerolipids. These findings support the idea that IRF5-deficiency in microglia leads to impaired intracellular lipid processing and homeostasis, which might contribute to microglial dysfunction and the observed remyelination failure.

Since IRF5-deficient microglia showed an increase in the accumulation of cholesterol esters (CEs), we next investigated whether the accumulated myelin-derived cholesterol was released into the extracellular space via specific transporters or converted to CE by cholesterol acyltransferase and then stored in intracellular lipid droplets (LDs). We assessed cholesterol storage after LPC-induced demyelination (14 days post-injection) using Oil Red O^+^ (ORO) staining, which selectively stains neutral lipids [57]. Consistent with our previous findings, we detected an increase in the number and size of lipid droplets (LDs) in the lesions of *Irf5*^-/-^ mice (Fig. 7D). Since defective cholesterol processing in microglia is associated with the pathological formation of dense crystals [58], we hypothesized that *Irf5*^-/-^ mice might exhibit this feature. Indeed, reflection microscopy showed a higher accumulation of cholesterol crystals in the lesions of *Irf5*^-/-^ mice compared to WT animals (Fig. 7E). Because phosphatidylcholines were slightly elevated at the periphery of the lesions in *Irf5*^-/-^ mice, we also examined the accumulation of oxidized phosphatidylcholines (OxPCs) in LPC-demyelinated lesions. OxPCs are considered pro-degenerative factors in multiple sclerosis, usually processed and neutralized by microglia [59]. Despite the disruptions in *Irf5*^-/-^ microglial myelin phagocytosis and lipid metabolism, E06 staining showed no significant differences in OxPC levels between WT and *Irf5*^-/-^ lesions (Fig. 7E). Thus, we concluded that the primary alteration in lipid metabolism in *Irf5*^-/-^ microglia is an overload of cholesterol esters, leading to an aberrant accumulation of cholesterol crystals and lipid droplets in the lesions.

### Facilitating cholesterol transport improves EAE symptoms in *Irf5*^*-/-*^ mice

**Fig. 8:**
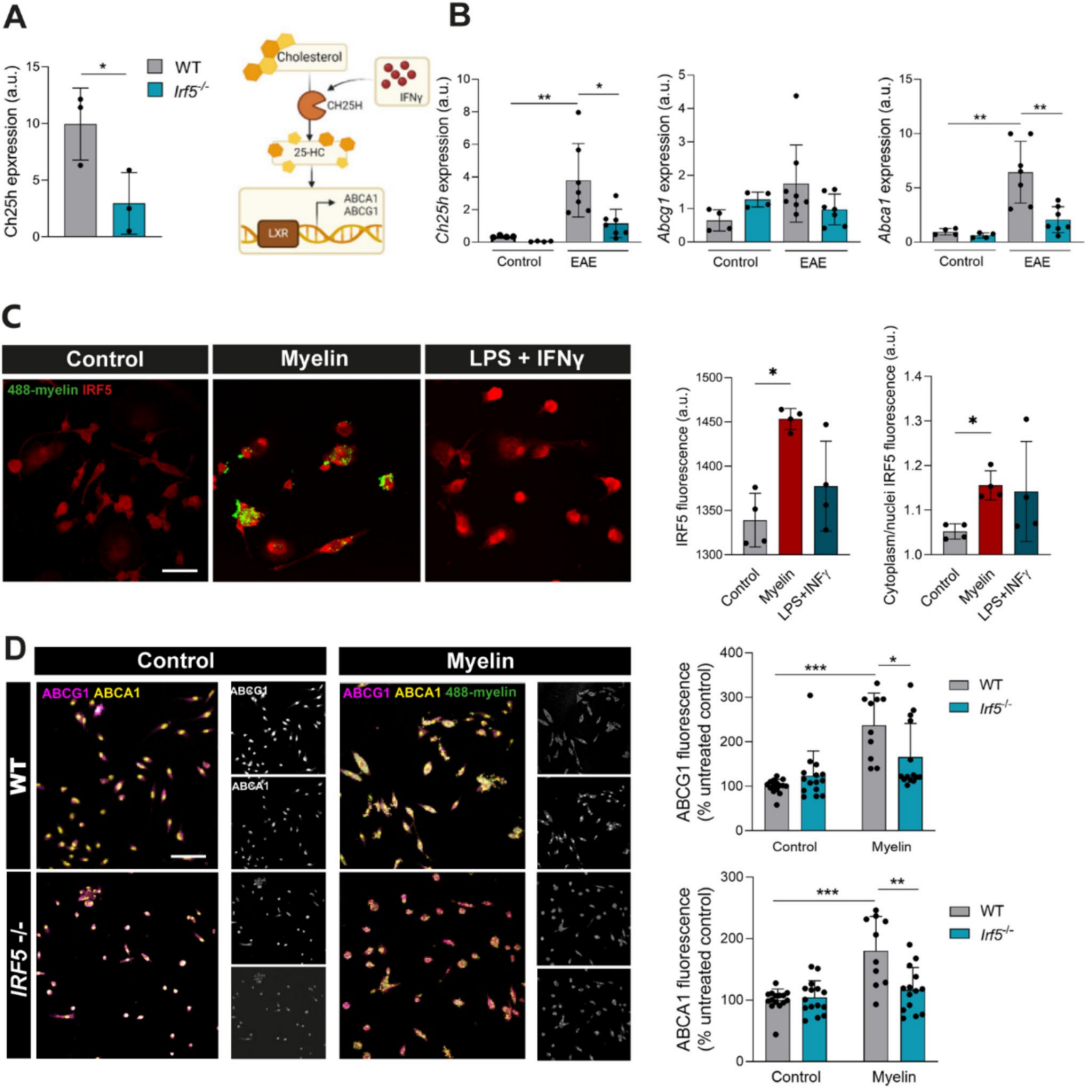
IRF5 deficiency alters *ch25h* expression and cholesterol transport. **A** *Left,* gene expression of cholesterol 25-hydroxylase (*ch25h*) in WT and *Irf5*^-/-^ microglia isolated by FACS (Fig. 4A). *Right*, scheme of the metabolic role of ch25h and 25-hydroxy-cholesterol (25HC) in lipid homeostasis. **B** Relative expression of *ch25h* and cholesterol transporters *Abca1* and *Abcg1* in total mRNA isolated from spinal cord of control and chronic EAE mice (*n* = 4–7). **C** *Irf5* expression in cultured microglia in basal condition and after exposure to myelin (25 μg/ml, 48h) or LPS plus IFNγ (10 ng/ml and 20 ng/ml respectively) (*n* = 4 independent experiments). Scale bar = 20 μm. **D** ABCA1 and ABCG1 expression in WT and *Irf5*^-/-^ microglia in basal conditions and after myelin exposure (25 μg/ml, 48h) (n= 5–7 independent experiments in duplicate). Scale bar = 50 μm. *p < 0.05, **p < 0.01, ***p < 0.001

Since cholesterol is a major component of myelin, myelin-derived cholesterol becomes a critical factor in regulating the regenerative response of microglia. Cholesterol cannot be degraded within cells and must be exported to the extracellular environment via cholesterol transporters such as ABCA1, ABCG1 and ApoE or esterified by acyl-CoA:cholesterol acyltransferase for storage in LDs, leading to the formation of foamy microglia/macrophages [60, 61]. We therefore checked whether the mechanisms involved in cholesterol export were altered in *Irf5*^-/-^ microglia, this leading to the observed aberrant accumulation of myelin-derived cholesterol observed. Interestingly, we found that the interferon-responsive gene *ch25h*, encoding the enzyme cholesterol 25-hydroxylase that catalyses cholesterol to generate 25-hydroxycholesterol, was downregulated in *Irf5*^-/-^ microglia (Fig. 8A). Moreover, although *ch25h* expression was significantly increased at EAE chronic phase (Fig. 8B), *Irf5*^-/-^ mice showed lower levels of *ch25h* transcripts than the WT mice at that condition (Fig. 8B). As 25-hydroxicholesterol is an agonist of nuclear receptor LXR regulating the transcription of cholesterol transporters [62] (Fig. 8A), we hypothesized that the expression of *Abca1* and *Abcg1* cholesterol transporters could also be altered in *Irf5*^-/-^ mice and microglia. Indeed, the expression of *Abca1* was significantly lower after EAE in *Irf5*^-/-^ mice (Fig. 8B).

We then tested this signaling pathway in microglia *in vitro*. The exposure of microglia to an excess of myelin (25 µg/mL; 48 h) induced an increase in the expression of IRF5 and its translocation to the nucleus (Fig. 8C), an effect similar to that observed in the presence of a pro-inflammatory stimulus, based on LPS and IFN γ(10 ng/mL and 20 ng/mL, respectively). As expected, myelin exposure also induced increased expression of ABCA1 and ABCG1 cholesterol transporters in WT microglia, while this upregulation was not present in *Irf5*^-/-^ microglia (Fig. 8D). Thus, deficient transport of the cholesterol could lead to the aberrant accumulation of cholesterol in *Irf5*^-/-^ microglia.

**Fig. 9:**
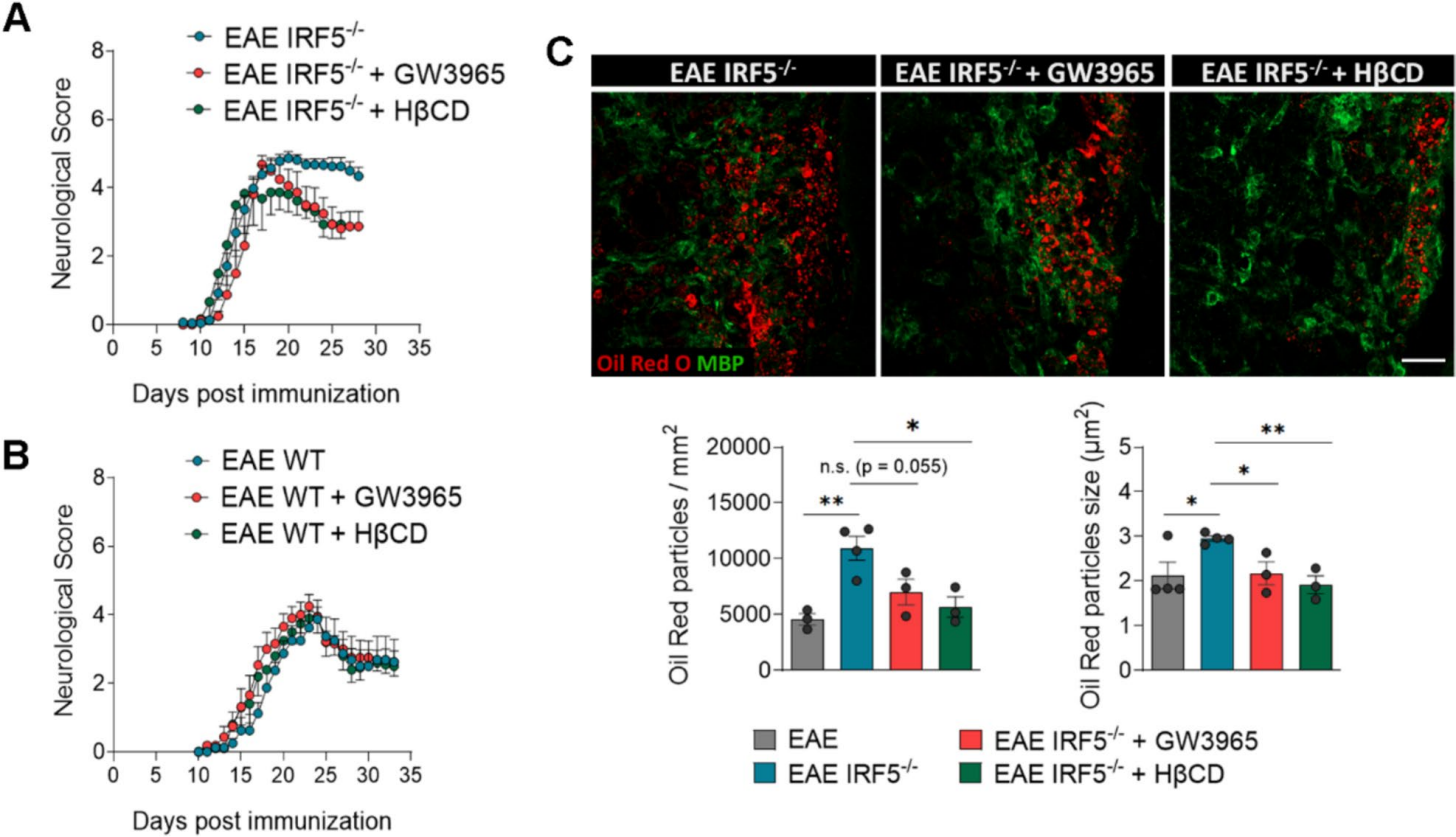
Treatment preventing CE accumulation reverses EAE exacerbated pathology in *Irf5*^-/-^ mice. **A** Neurological score of *Irf5*^-/-^ mice treated with saline, GW3965 (20 mg/kg; i.p.), an LXR agonist, and 2-hydroxypropyl-β-cyclodextrin (HβCD; 400 mg/kg; subcutaneous injection every 48h). Treatments started at 10 days postimmunization to avoid interfering with immune priming (*n* = 8–10). **B** Neurological score of WT mice treated with GW3965 and HβCD, as described before (*n* = 8). **C** Representative images of lipid droplets (Oil Red O staining) accumulation inside the EAE lesions in *Irf5*^-/-^ mice without treatment or treated with GW3965 and HβCD Histograms show the number and size of ORO^+^ particles in the lesions (*n* = 3–4). Scale bar = 25 µm. Data are presented as means ± SEM. Statistics were performed with Mann-Whitney U test (neurological score, **A**) and one way ANOVA (**B**)

Lipid droplet-accumulating microglia represent a dysfunctional and proinflammatory state that could be contributing to the exacerbated damage observed in *Irf5*^-/-^ after EAE. We therefore reasoned that facilitating cholesterol transport could improve EAE neurological symptoms. To test this hypothesis, we used two complementary pharmacological approaches: GW3965, a liver X receptor (LXR) agonist that reduces CE accumulation by upregulating the expression of ABCA1 and ABCG1 transporters [63]; and 2-hydroxypropyl-β-cyclodextrin (HβCD), which reduces intracellular cholesterol accumulation, notably in Niemann–Pick disease type C1 disorder [64]. We tested the impact of GW3695 and HβCD on EAE pathogenesis in *Irf5*^-/-^ mice. GW3695 (20mg/kg daily; i.p. injection) and HβCD (400mg/kg every 48 hours; subcutaneous injection) were administered from 10 post-immunization to avoid interfering with immune priming. Both GW3695 and HβCD led to a significant improvement in neurological symptoms in *Irf5*^-/-^ mice (Fig. 9A). In contrast, no effect of the drugs was observed in WT mice (Fig. 9B). Consistently, oil red staining showed a significant reduction in lipid droplets accumulation in demyelinated lesions (Fig. 9C).

Altogether, these results confirm the role of IRF5 in lipid metabolism in response to demyelination. The absence of this transcription factor disrupts myelin processing and leads to the formation of abnormal lipid structures, hindering proper regeneration.

## Discussion

Multiple sclerosis (MS) lesions often exhibit an accumulation of myelin debris and lipids, such as cholesterol, which could affect the regenerative response [52, 58, 65]. As a result, microglial lipid phagocytosis and processing following demyelination have become key areas of research aimed at promoting remyelination [9, 51, 66]. Our findings reveal a novel role for IRF5 in regulating lipid metabolism in microglia, alongside its well-established function in orchestrating immune responses. Specifically, *Irf5*^*-/-*^ mice exhibited impaired myelin processing and lipid homeostasis in response to demyelination, which hindered remyelination. However, promoting cholesterol transport was able to reverse these effects.

Polymorphisms in IRF5 have been linked to a heightened risk of developing autoimmune diseases [67]. IRF5 is determinant to promote disease development in a murine model of lupus erythematosus autoimmune disease [68]. Similarly, our data suggest that IRF5 has a significant impact on adaptive immune priming, as *Irf5*^-/-^ mice experienced a delay in EAE onset. This aligns with previous studies showing that other IFN transcription factors are involved in EAE development. Thus, *Irf1*^*-/-*^*, Irf3*^*-/*-^, or *Irf8*^-/-^ mice exhibit either reduced EAE pathogenesis or, in the case of IRF8, complete resistance to EAE [40, 69, 70]. Surprisingly, despite the initial delay in immune priming, *Irf5*^-/-^ mice exhibited a more severe EAE pathogenesis during the chronic phase. This exacerbated damage and remyelination failure were also observed in a model in which demyelination is not immune-mediated, such as lesions induced by lysophosphatidylcholine (LPC) injections into the spinal cord, suggesting that the IRF5-related mechanisms impacting remyelination involve CNS parenchymal microglial cells—the only cells in the CNS expressing *Irf5*. [12, 71]. However, the impact of *Irf5* in other infiltrating immune cells could not be entirely excluded in the EAE model. These findings indicate that IRF5 has a beneficial role in regeneration and remyelination, consistent with studies suggesting that inflammatory activation of myeloid cells is crucial for these processes [72, 73]. Indeed, both TLR responses and MyD88- mediated pathways, which are directly linked to IRF5, are significant for remyelination [74].

Interferon signaling is known to play a role in regulating microglial activity in MS. A type I interferon gene signature in microglia, marked by the expression of antiviral genes such as chemokines, signaling proteins, enzymes, and transcriptional regulators, has been identified in demyelinating lesions [75]. Notably, a recent study found a similar type I interferon-responsive microglia subset that engulfs neurons in the developing mouse cortex, demonstrating a physiological role of a canonical antiviral immune pathway in brain development [76]. Mice lacking the obligate receptor for the IFN-I response, Ifnar1, displayed phagolysosomal dysfunction [76], suggesting that interferon signaling may be important for the degradation of phagocytosed neurons. In the EAE model, *Ifnar1* knockout mice show exacerbated neurological symptoms [77] and greater accumulation of myelin debris in lesions [77, 78]. IFN signaling is also necessary to induce microglial turnover and recycling, which is crucial for remyelination the LPC model [79]. Thus, interferon signaling in microglia appears to have pleiotropic, context-dependent effects in demyelinating disorders.

Our transcriptomic and lipidomic analyses identified IRF5 as a key transcriptional regulator of lipid endocytosis and degradation in microglia. One potential link between IRF5 and lipid homeostasis, though not the only one, could be the *ch25h* gene, which encodes the enzyme cholesterol 25-hydroxylase. In the CNS, *Ch25h* is predominantly expressed by microglia [31], is upregulated under inflammatory conditions [80], and is part of the gene set expressed by disease-associated microglia (DAM) that remain chronically upregulated in various neurodegenerative diseases [81, 82]. *Ch25h* catalyses the conversion of cholesterol to 25-hydroxycholesterol, which activates liver X receptors (LXR), driving the transcription of genes involved in cholesterol homeostasis and efflux, such as the *Abca1* and *Abcg1* transporters [62]. Therefore, the downregulation of *ch25h* observed in *Irf5*^-/-^ mice may contribute to the reduced expression of cholesterol transporters and the accumulation of cholesterol in microglia.

LD-accumulating microglia represent a dysfunctional state in the aging brain [83], and a similar phenotype has been described in microglia lacking TREM2 [52, 58, 65]. These cells abnormally enhanced LD production when challenged with an excess of myelin, leading to pathogenic events such as the formation of cholesterol crystals, which can block remyelination [52, 83, 84].

Interestingly, recent evindence suggest that LD biogenesis in phagocytes could play a role in promoting remyelination [84]. One of the factors that may determine whether LDs have a beneficial or detrimental role is their proper processing. Indeed, increasing lypolisis-mediated LD turnover by targeting perilipin-2, a major LD surface proteins has recently been proposed as a possible theraupetic target to promote regeneration [85]. This hypothesis is supported by the aberrant accumulation of cholesterol crystals in LDs in aged phagocytes, suggesting a faulty cholesterol metabolism that leads to maladaptive pro-inflammatory responses and failure in remyelination after LPC injections [58]. Similarly, in *Irf5*^-/-^ microglia/macrophages, cholesterol crystal accumulation was observed following LPC treatment, and facilitating cholesterol efflux reversed the increased neurological symptoms in the EAE model. Overall, these results suggest that the defective cholesterol processing in *Irf5*^*-/-*^ mice hinders the anti-inflammatory, pro-regenerative functions of microglia/macrophages. Also, disruptions in lipid efflux could potentially dampen the capacity to recycle lipids for remyelination [51].

*Irf5*^-/-^ microglia also exhibited downregulation in genes related to GTPases signaling, postulating these pathways as potential targets for this transcription factor. GTPases play a critical role in modulating actin cytoskeleton remodelling, and are known to affect microglial migratory and phagocytic capacity [45, 47, 86]. Moreover, the loss of Rho small GTPases in microglia has been associated with pro-degenerative, excitotoxic effects [87]. The altered phosphatidylinositols (PI) metabolism observed in *Irf5*^-/-^ microglia, as detected by LC/MS, could contribute to the impaired GTPase signaling, given that PIs, along with other phospholipids, play an active role in these signaling pathways in microglia, as well as in some other intracellular processes [88]. Unlike *Irf8*^-/-^ macrophages, which exhibit reduced migratory capacity toward lesion epicentres after spinal cord injury [48], we did not find direct evidence for reduced motility in *Irf5*^-/-^ macrophages after demyelination. However, the disrupted phagocytic response to myelin debris could be a secondary effect of altered GTPase signaling, in addition to intracellular lipid overload. It's noteworthy that recent studies have shown that IRF5 is involved in macrophage migration into the adventitia in abdominal aortic aneurysm [89], indicating its broader role in cell migration and suggesting further exploration into its potential impact on microglial motility and function in other contexts.

## Conclusions

The findings from this study suggest a novel role for IRF5 in priming microglia for remyelination, primarily through its modulation of lipid degradation and homeostasis. While the absence of IRF5 might offer some benefits during the early stages of demyelination, its expression tends to decrease in microglia as the disease progresses. Therefore, boosting IRF5 activity in microglia could be a promising therapeutic approach to facilitate effective myelin clearance and to promote pro-regenerative events at later stages of the disease.

## Supplementary Information

Below is the link to the electronic supplementary material.

Supplementary file1 (DOCX 2228 KB)

## Data Availability

The transcriptomic datasets generated during the current study will be made accessible in public repository NCBI upon publication. The lipidomic datasets generated will be available from the corresponding author on request.
