# Supplementary material for: Microglia regulate myelin clearance and cholesterol metabolism after demyelination via interferon regulatory factor 5
Source: Cell Mol Life Sci. 2025 Mar 26;82(1):131. doi: 10.1007/s00018-025-05648-2 (PMC11947375; doi:10.1007/s00018-025-05648-2)
Supplement: Supplementary file 1 — Supplementary file1 (DOCX 2228 KB) [file 18_2025_5648_MOESM1_ESM.docx]

**SUPPLEMENTARY FIGURES LEGENDS**

**
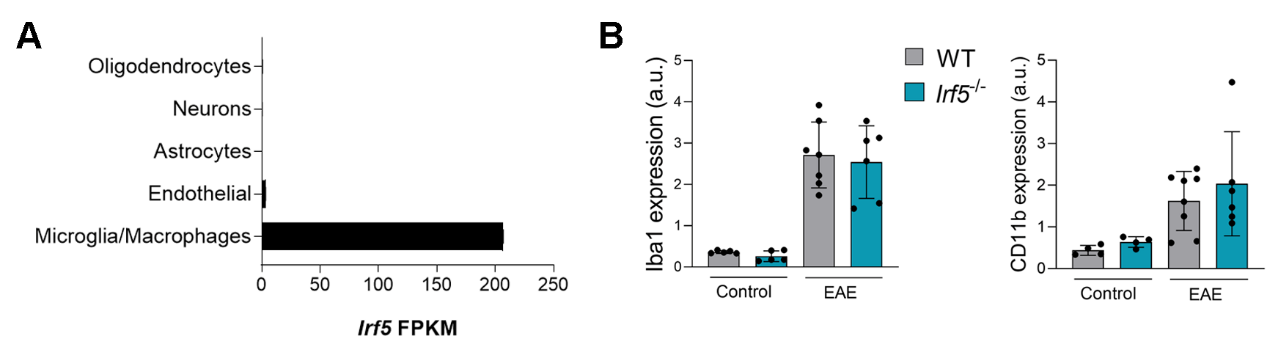
**

**Figure S1.** (**A**) Analysis of *Irf5* mRNA expression in CNS parenchymal cells from scRNAseq database [77]. (**B**) Relative expression of microglial markers *Iba1* and *CD11b* in total RNA isolated from the spinal cord of WT and *Irf5*^-/-^ mice at EAE chronic phase (*n* = 4-7).

**
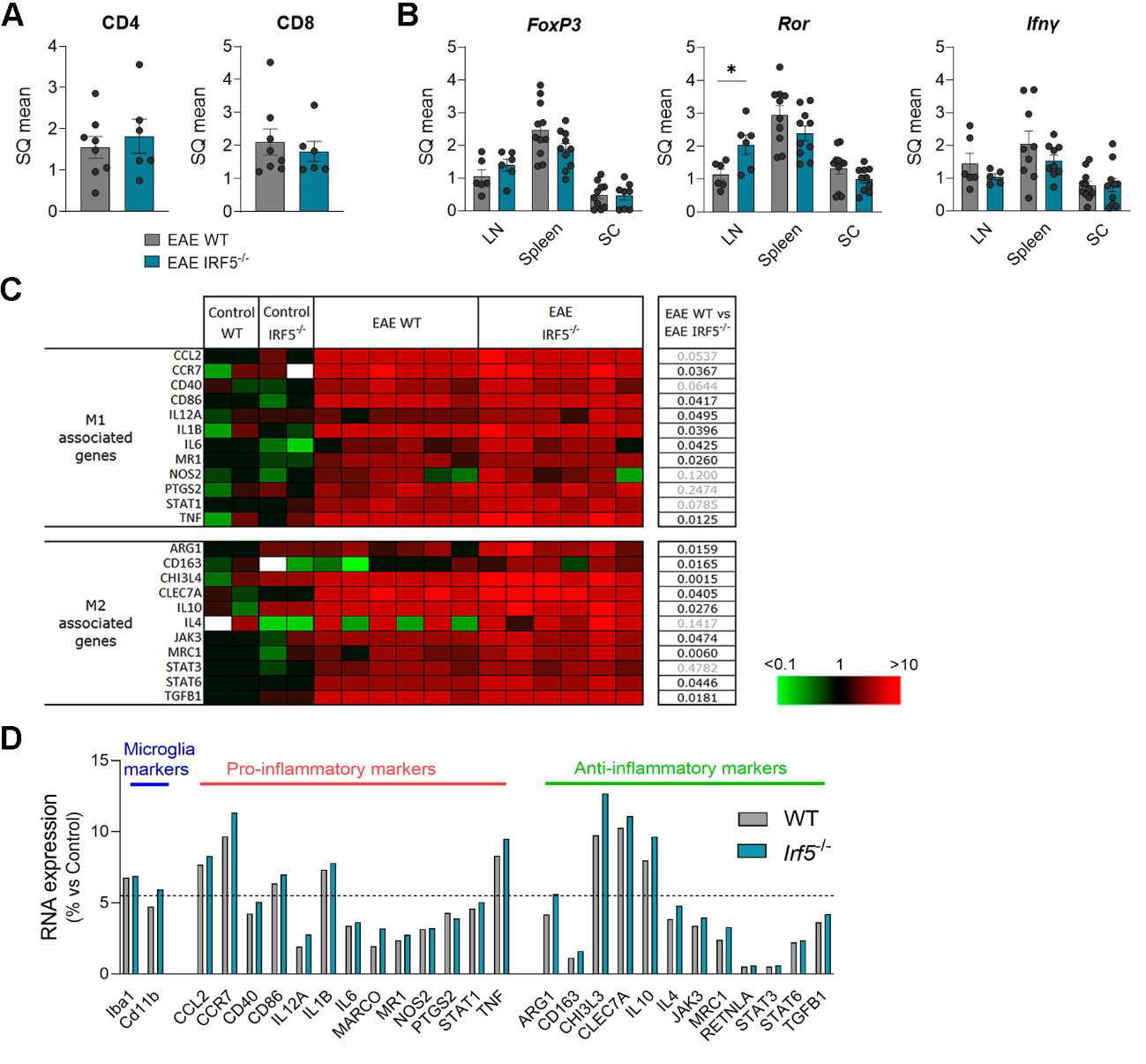
Figure S2. IRF5 deficiency has no impact on immune response. (A)** Relative mRNA expression of *Cd4* and *Cd8* in the spinal cord of WT and *Irf5*^-/-^ mice at EAE chronic phase (*n* = 6-8). (**B**) Relative mRNA expression of *Foxp3* (Tregs), *Ifnγ* (Th1) and *Ror* (Th17) in lymph nodes (LN), spleen and spinal cord of WT and *Irf5*^-/-^ mice at EAE chronic phase (*n* = 6-8). (**C**) Heatmap showing significant changes in pro-inflammatory and anti-inflammatory mRNA expression in total RNA isolated from the spinal cord of WT and *Irf5*^-/-^ mice at EAE chronic phase (*n* = 6). Tables indicate statistical significance between EAE WT and EAE *Irf5*^-/-^ mice. Data were analyzed by unpaired Student’s t-test. * p<0.05.

(**D**) Expression of pro-inflammatory and anti-inflammatory genes, as described in C, relative to the expression of microglial markers *Iba1* and *CD11b* in total RNA isolated from the spinal cord of WT and *Irf5*^-/-^ mice at EAE chronic phase (*n* = 4-7).

**
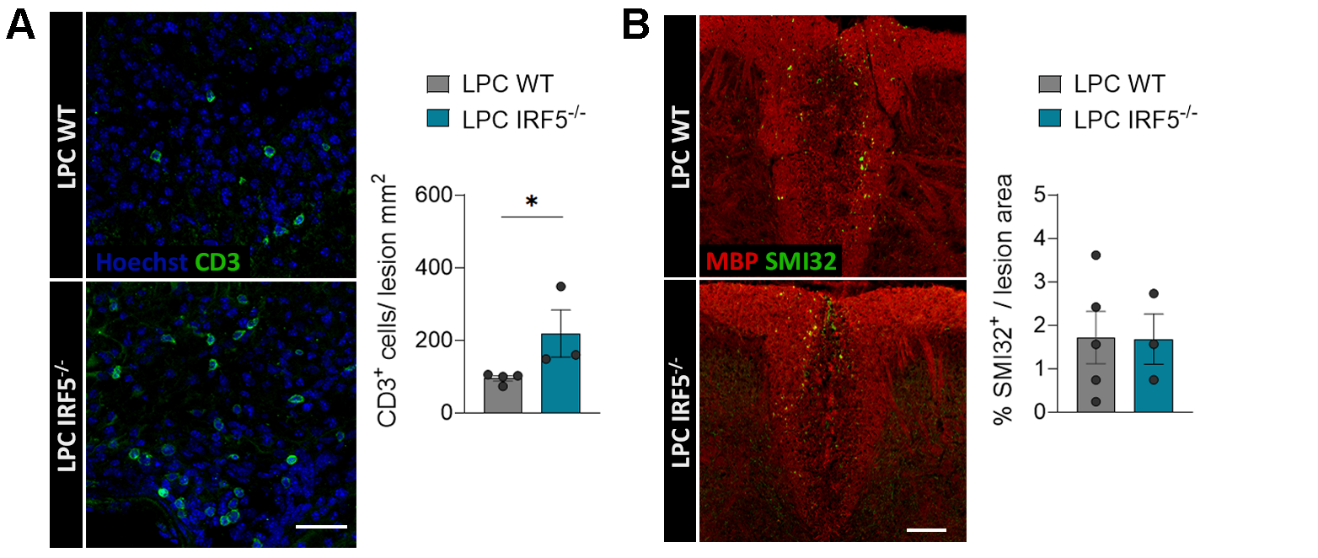
**

**Figure S3. IRF5 deficiency alters T cells response after lysolecithin (LPC)-induced demyelination.** (**A, B**) Spinal cord sections of wild type (WT; n = 5) and *Irf5*^-/-^ (n = 6) mice 14 days after LPC injection immunolabelled for CD3 T cell marker (**A**) and for SMI32 and MBP (**B**), markers of axonal damage and myelin. Scale bars = 30 and 75 µm, respectively. Histograms shows means ± SEM (n = 3-5). Statistics were performed with Student’ t-test. **p* <0.05.

**
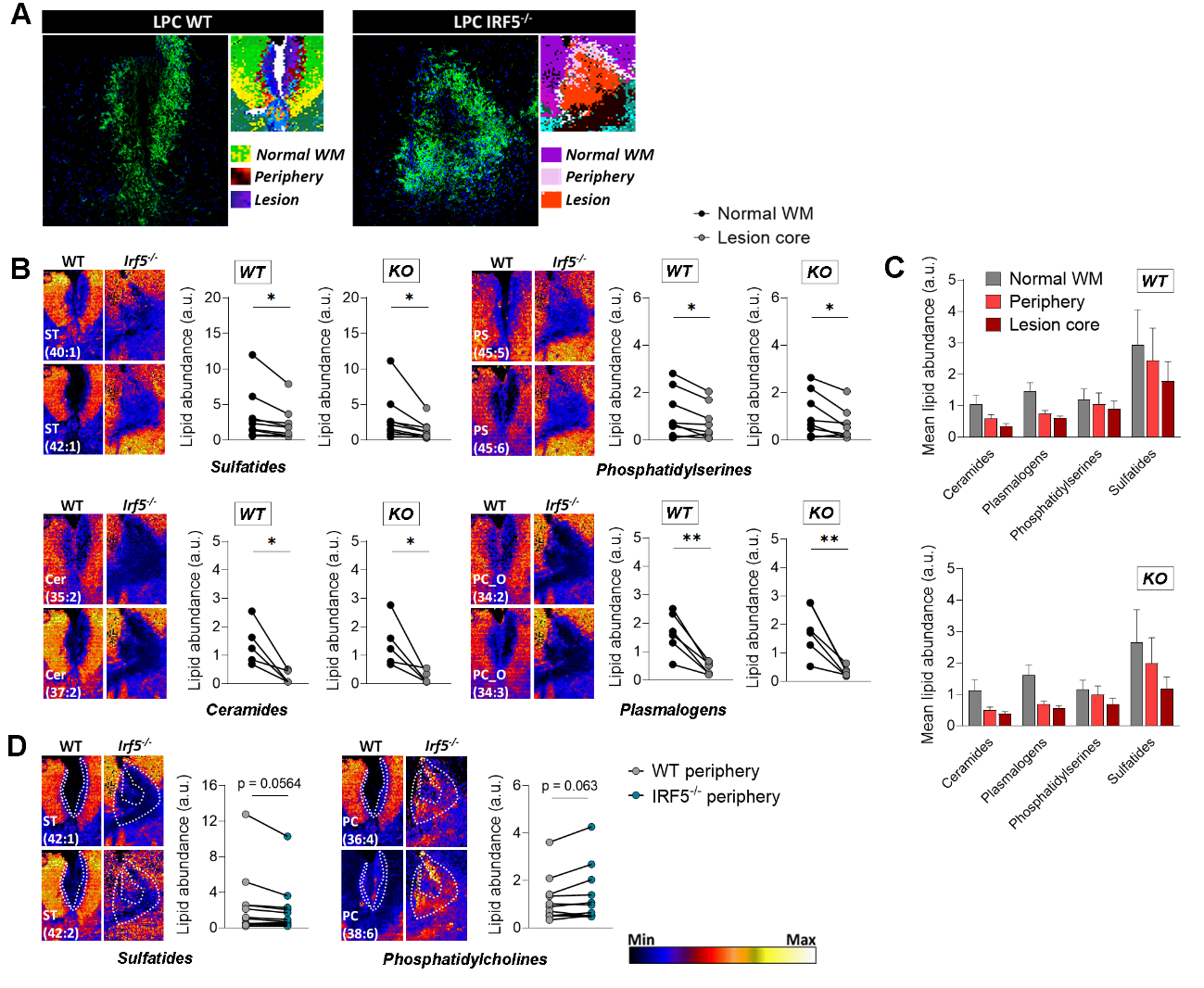
Figure S4**. **MALDI-IMS profiling of lipids in lysolecithin (LPC)-induced demyelination**. (**A**) Representative immunohistochemical images of LPC lesions (14 dpi), identified on the basis of Iba1 accumulation as well as MBP loss (not shown), and parallel lipid segmentation obtained of these lesions, following k-means clustering method. Of note, there are different lipid profiles corresponding to the lesion core, the periphery and the normal WM. (**B**) Representative MALDI mass spectrometry imaging (MSI) maps of major sulfatides (ST; 40:1 and 42:1), phosphatidylserines (PS; 45:5 and 45:6), ceramides (Cer; 35:2 and 37:2) and plasmalogens (PC_O; 34:2 and 34:3) in the WT and *Irf5*^-/-^ lesions. Histograms shows the difference of abundance of different families of lipids in the normal white matter (WM) and the lesion core of WT and *Irf5*^-/-^ mice. Each point in the graphs represent a single lipid species analyzed by MALDI. (**C**) Heatmaps showing the mean lipid concentration of the different ceramides, plasmalogens, phosphatidylserines and sulfatides identified in the normal WM, the periphery and the lesion core in WT and *Irf5*^-/-^ mice. (**D**) Representative MALDI mass spectrometry imaging (MSI) maps of major sulfatides (ST; 42:1 and 42:2) and phosphatidylcholines (PC; 36:4 and 38:6) in the WT and *Irf5*^-/-^ lesions. Histograms shows the difference of abundance of different families of lipids in the WT and *Irf5*^-/-^ lesion periphery. Lipid abundance scale for MALDI-MSI images is present in the bottom right corner of the figure. Data are presented as means for every lipid species from n = 5 different mice. *p < 0.05, **p < 0.01.
